# Influence of 3D Printing Direction in PLA Acoustic Guitars on Vibration Response

**DOI:** 10.3390/polym15244710

**Published:** 2023-12-14

**Authors:** Álvaro Burgos-Pintos, Francisco Fernández-Zacarías, Pedro F. Mayuet, Ricardo Hernández-Molina, Lucía Rodríguez-Parada

**Affiliations:** 1 Department of Mechanical Engineering & Industrial Design, Faculty of Engineering, University of Cadiz, Av. University of Cadiz 10, 11519 Puerto Real, Spain; alvaro.burgospintos@alum.uca.es (Á.B.-P.); francisco.fernandez@uca.es (F.F.-Z.); ricardo.hernandez@uca.es (R.H.-M.); lucia.rodriguez@uca.es (L.R.-P.); 2 Acoustic Engineering Laboratory, University of Cadiz, 11519 Cadiz, Spain

**Keywords:** additive manufacturing, FFF, PLA, acoustic guitar, product design, vibration testing, parameterisation, simulation

## Abstract

The design of musical instruments is a discipline that is still carried out in an artisanal way, with limitations and high costs. With the additive manufacturing technique, it is possible to obtain results for the generation of not only electrical but also acoustic instruments. However, it is necessary to generate a procedure to evaluate the influence of the process on the final result of the acoustics obtained. This study focuses on investigating the relationship between the construction of acoustic guitars and their final sound. The reinforcement structures at the top of the instrument are analysed, as well as how this design affects the vibratory behaviour of the top in the first five vibratory modes. Specifically, this article presents a procedure for the design of customised acoustic guitars using additive manufacturing through parametrisation and a vibrational analysis of the designed tops using finite element (FEA) and experimental physical tests, in order to develop a methodology for the study of stringed instruments. As a result, an 11% increase in the high-frequency response was achieved with a printing direction of +45°, and a reduction in the high-frequency response with ±45°. In addition, at high frequencies, a relative error of 5% was achieved with respect to the simulation. This work fulfils an identified need to study the manufacture of acoustic guitars using polylactic acid (PLA), and to be able to offer the musician a customised instrument. This represents a breakthrough in the use of this manufacturing technology, extending its relationship with product design.

## 1. Introduction

Currently, industrial design is gaining importance owing to the increasing demand from users for the creation of personalised designs. In the music sector, research is oriented towards the creation of new designs through the search for new geometries with greater complexity, thus offering a wider spectrum and possibilities in different aspects, such as function, sound and aesthetics [1,2]. In this respect, additive manufacturing (AM) allows designs with novel geometries and greater complexity than conventional manufacturing methods [3]. An example of this is the case of flutes designed with non-traditional geometries using AM, which are manufactured using FFF, achieving a similar acoustic result, combining parts of the traditional instrument with printed parts, generating the same sound quality as the traditional flute [4]. Thanks to this technology, it was possible to print the different components of the instrument, and to generate a design with specific printing parameters for an easy assembly of the instrument. The importance of the creation of thick walls was observed, which provided mechanical resistance to pressure, which is fundamental for the correct functioning of the flute. With regard to air-instrument components, the work of Bacciaglia et al. [5] shows the differences in the use of FDM with SLA, as FDM does not provide the best acoustic response for a trumpet mouthpiece in this case. Instead, the possibilities of such fabrications for the generation of new geometries are shown. Furthermore, Marano et al. simulated and designed a trumpet with a variation of radii in its structure, depending on the desired sound result, which is a great example of combining CAD tools and 3D printing, providing rapid fabrication and design modification [6]. On the other hand, the electric violins of the brand 3D Varius [6] are clear examples of the results of research in instrument customisation and manufacturing by means of AM [2]. In particular, these products feature user-customised designs based on ergonomics and aesthetics [7].

As described above, customised electric guitars made by AM can also be found on the market currently. These have a dual purpose: to improve ergonomics and to create a fully customised aesthetic appearance [8,9]. There is potential for innovation in both these areas, thanks to the possibility of creating complex geometries that would not be possible with conventional manufacturing techniques [10,11,12]. However, in these electric guitars, the acoustics are not affected by the change in geometry [13,14]. In this respect, the acoustic guitar represents a paradigm for the creation of customised products by AM. This is due to the close relationship between the design of the instrument and the sound result to be obtained [15,16].

In the field of acoustic guitars, taking into account the existing body of research in relation to the acoustic behaviour of the instrument and its construction according to the most relevant luthiers, we must highlight the most important area of the acoustic guitar for the generation of sound: the top plate. Depending on its construction, which is mainly influenced by its reinforcement structure, guitar bracing, the instrument acquires one sound or another; that is, its behaviour at high or low frequencies and high or low notes, varies, defining a particular sound in each case [17,18,19,20,21,22,23,24].

This sound result is observed in the family of instruments created by MONAD, which generates a series of violins, as well as cellos with organic geometries, whose sounds are completely different from traditional versions. The aim of this creation is to demonstrate the possibility of broadening the sound spectrum of the instruments, generating complex and changing geometries depending on the aesthetics of the instrument [25,26].

At the same time, additive manufacturing currently provides good sound quality, which means an advance in the future customisation of the instrument, since traditional manufacturing does not facilitate this adaptation to users, but the use of advanced technologies, such as 3D scanning, or the use of finite element simulations, makes it possible to design in a more creative way, as it is possible to visualise or establish the final behaviour of the designed instrument, without the need for costly or complex manufacturing due to the geometries generated. It is true that functionally similar results can be achieved by conventional methods, such as flexible manufacturing systems (FMS) or manufacturing cells. However, from economic and energy points of view, this would require a much higher investment than the aforementioned manufacturing technology. In addition, it should be noted that the use of technologies other than AM can have a negative impact on the environmental performance of the process [27,28,29].

An important aspect to highlight in product design, with respect to the use of AM as the main technology, is the combination of materials. This has been explored in studies such as that by Li et al. [30], in which different printing directions are discussed, at the time of manufacturing, and how this can be a resource when used in combination with another material, thus increasing the properties of the final piece. This work, like those carried out by Ferdousi et al. [31] and Nugraha et al. [32], shows great potential for the creation of a methodology that combines mathematics with FFF, which is becoming important and relevant to the work presented. In addition, in the music and instrument manufacturing sectors, it is essential to provide new geometries that can provide new information to this sector.

As mentioned above, a great deal of work is underway in which AM is combined with custom design. In this sense, there is an opportunity to bring this technology closer to the music sector, more specifically in the creation of acoustic guitars by means of such manufacturing. To this end, this work analyses the acoustics of the instrument, creating a methodology that broadens the knowledge of the vibratory behaviour of the PLA material in the design of this musical instrument. The vibration analysis was carried out by means of physical experiments and simulations, which provided a visualisation of the vibration patterns generated in the first five vibration modes of the samples tested, as well as making it possible to analyse the effects of the variation of certain printing parameters on the vibration of the top plates [33,34,35,36]. The aim of this work is to make a comparison of the printing parameters with respect to the printing directions of the lids with their vibrational response.

## 2. Materials and Methods

The research carried out consisted of keeping the guitar shape and size constant, design of six types of harmonic top, and analysing the deformations caused and vibration patterns provoked by the different geometries. For this purpose, an experiment was carried out in which different harmonic specimens designed to obtain the vibrational behaviour of the 3D printed guitar tops were excited in the frequency range of 200 to 850 Hz.

The experiment carried out featured experimental tests of the first five vibration modes, selected on the basis of the five natural frequencies, as it is these that cause the whole harmonic top to resonate, according to previous studies [37,38]. In this way, the generated vibration patterns can be visualised, showing the areas of the greatest and least vibration of the harmonic cap created in each case. In addition, five 3D printing directions were analysed in the physical tests. In the specified frequency range, the main differences in the vibrational behaviour of an acoustic guitar soundboard can be observed according to previous works [24,27]. In the second phase, the results obtained in the experimental tests were validated using test specimens via simulations. A total of 60 printed test specimens were tested, as two specimens of each type were tested, and the dispersion of the results obtained from both was negligible, demonstrating similar responses for both specimens.

It should be taken into consideration that the simulation did not replicate the internal structure of each specimen because one of the objectives to be analysed in this work is to observe the effects of the changes in the variables in the construction of the different specimens, and the vibration response associated with each of them [39,40,41,42,43].

### 2.1. Guitar Design and Parameterisation

**Figure 1:**
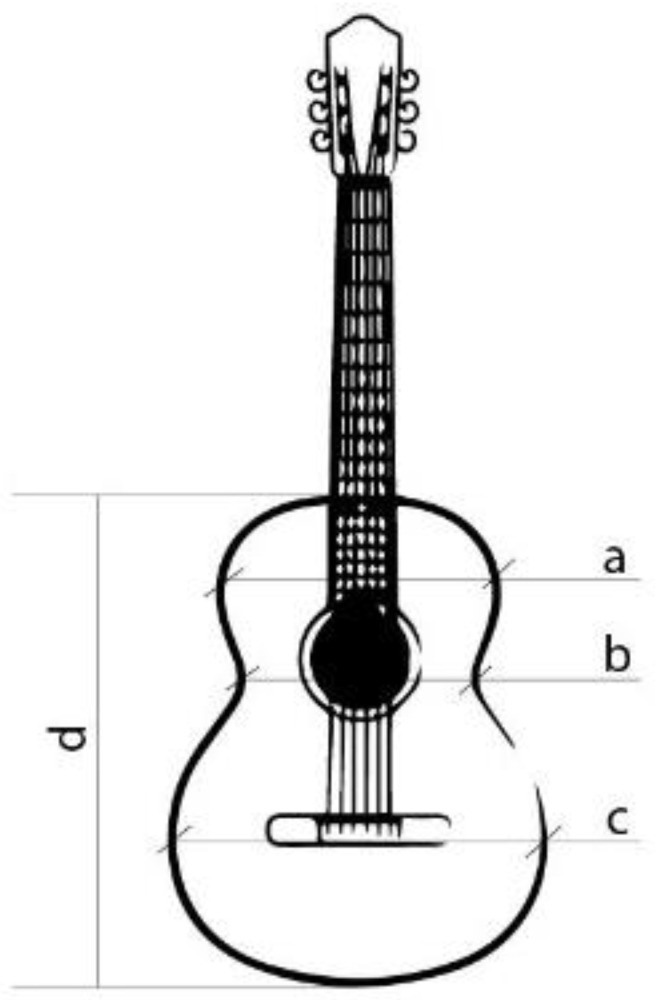
Parts of the top plate. (a) Upper bout, (b) waist, (c) lower bout, (d) overall length.

**Figure 2:**
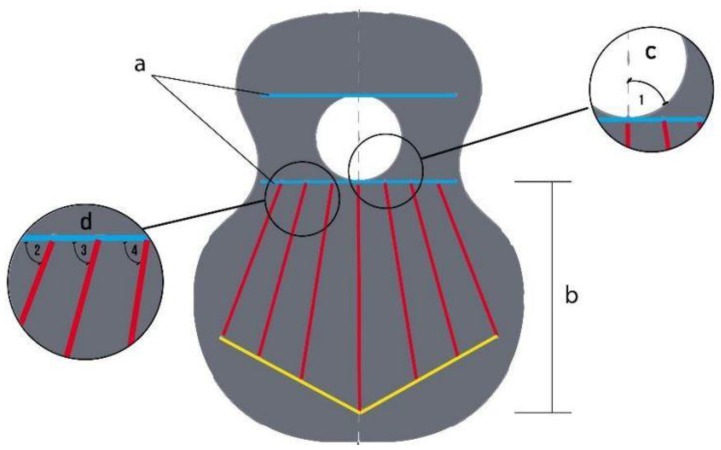
Parameterisation of guitar bracing: (a) harmonic rods, (b) fan distribution (c) angle 1 and (d) angles 2, 3 and 4.

For the design and parameterisation of the wailing, three main parts of a top plate were determined and differentiated (Figure 1). The upper bout is located at the top of the soundboard, closest to the neck of the guitar (Figure 1a). The waist is a critical area of the guitar, as it separates the upper and the lower bout and is an area of high stress for the instrument (Figure 1b). Finally, the lower bout was the area where most of the vibrations were produced when playing the instrument (Figure 1c). In this area, the bridge of the guitar, which transmits the vibration of the strings to the top, must resist the tension of the strings while respecting the elasticity necessary for the top to vibrate correctly. On the other hand, it is in these three parts, Figure 1a–c, that the vibration is provoked owing to the design of the bracing, thus generating the sound of the guitar [44]. Furthermore, in this work, two zones that affect the acoustics are also denoted: the right zone of the first three strings, or thinner strings, and the left zone of the last three strings of the instrument with the largest diameter, as shown in Figure 2.

In this experimental procedure, soundboards were designed and parameterised for AM, taking as a reference the guitar bracing created by Antonio de Torres, which is a basic structure in the construction of acoustic guitars [45]. This luthier designed a reinforcement structure, or guitar bracing, for the soundboard, which serves as the basis for the construction of derivative guitars because it provides a balanced sound [43].

According to Antonio de Torres’s guitar bracing, the minor lobe and waist lobe are separated by harmonic rods, which have a rectangular profile with a greater height with respect to the fan located in the major lobe, whose profile is square with a shorter side height Figure 2a.

In order to be able to parameterise the arrangement of the guitar bracing, and to observe the effect it has on its vibration, four main angles were distinguished. Angle 1 corresponds to the inclination of the second harmonic rod under the soundhole, and angles 2, 3 and 4 correspond to the inclination of the guitar bracing that makes up the fan of the main lobe.

**Figure 3:**
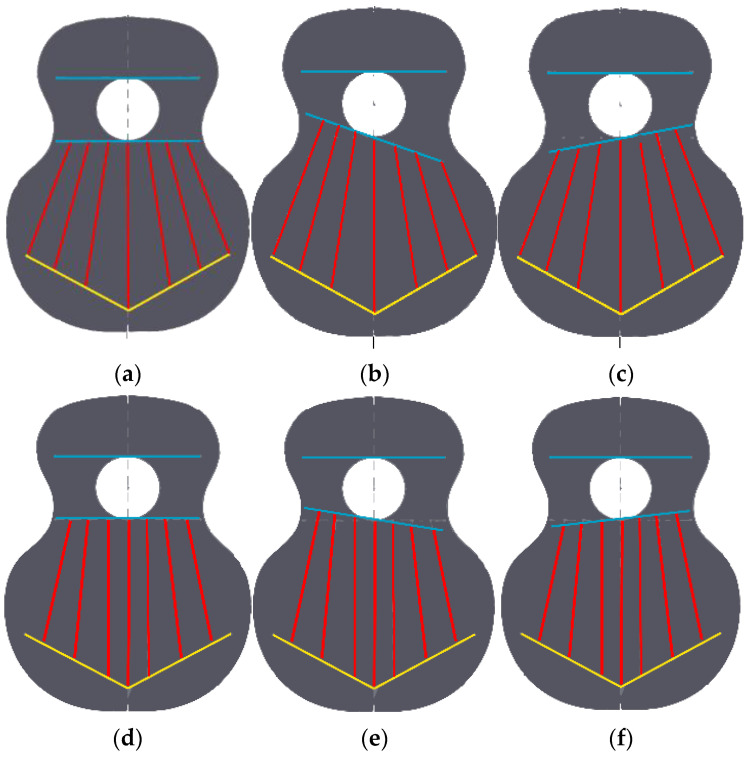
Parameterised covers: (**a**) T01 angle 1 with 90°, (**b**) T01 with angle 1 with 99°, (**c**) T01 with angle 1 with 81°, (**d**) T02 angle 1 with 90°, (**e**) T02 angle 1 with 99°, (**f**) T02 angle 1 with 81°.

Based on the above, six parameterised top plates were designed, as shown in Figure 3. Specifically, two fan designs were made, which differed in the initial inclination of each of the rods or reinforcements, angles 2, 3 and 4 of the fan distribution (Figure 2d). To carry out this parameterisation, the computer-aided-design program Solidworks 2019 SP5.1 (Dassault Systems) was used, in which simulation tests and frequency analysis can also be carried out [46]. However, the only variation in each of the three variants of the designs is angle 1, with respect to the vertical line. That is, the inclination of the horizontal line corresponding to the lower harmonic rod (Figure 2) and waist line (Figure 1) is modified. Three angles of position with respect to angle 1 were studied: 90° is the neutral placement, which corresponds to Figure 3a,e, 99° is the placement for low frequencies, which corresponds to Figure 3b,e, and 81° is the placement for high frequencies (Figure 3c,d).

The first design studied, T01, corresponds to the traditional fan layout shown in Figure 3a, according to [45]. It was defined by inclinations of 69°, 75° and 84°, taking the horizontal axis as a reference, corresponding to angles 2, 3 and 4, respectively. The second design, T02, had a fan-shaped distribution with variations in angles 2, 3 and 4 with respect to the horizontal (Figure 3d). The inclinations were 78, 84 and 90°, respectively.

### 2.2. Creation of Test Tubes

The material used in this test was a PLA filament with a diameter of 1.75 mm (Esun brand). The printer used for the creation of the specimens was Ender 3 Pro (Hong Kong, China), which has sufficient printing dimensions for the creation of the specimens tested in this study.

The mechanical properties of the material were obtained from the supplier in accordance with the quality standards ISO 9001–Quality Management Systems [47], REACH Regulation, RoHS Certification Services and EN 13432-2000 [48].

These properties were reproduced in the FEA simulation.

For the experimental tests, an Innova ALT/IN35 loudspeaker (Madrid, Spain) was used, the driver of which is the MKS 78F-076G (3 Omh-5 W), thanks to which the frequency sweep is transmitted to the different specimens, by means of a plastic tube attached from the speaker to the surface of the lid. The arrangement of the elements in the experiment is shown in more detail in later sections. In addition, for collection of further data for the top plate vibration, a wintact WT63B hand-held vibrometer (Shenzhen,China) was used, which covers measurement ranges from 0.1 to 199.9 and has the measurement ranges and accuracy detailed in Table 1.

Five printing directions were evaluated (Table 2). Specifically, directions of 0°, 90°, +45°, −45° and ±45° were evaluated. The remaining manufacturing parameters were kept constant in all prints. A 100% infill, 60 mm/s speed and 0.2 mm layer height were used, according to [49]. The nozzle used was 0.4 mm in diameter. During the printing processes of the different lids, their correct manufacture was analysed and checked. The dimensions of each of the tops with respect to the dimensions established for the model were corroborated. The correct thickness of the lid and the heights of the rods that made up the guitar bracing were taken into account.

It should be noted that wooden top plates were manufactured in the beta direction, perpendicular to the bridge position. For this reason, the printing direction was an important factor; therefore, in this research, we sought to analyse the impact of the variation in this 3D printing variable. This work does not take into account the response of the manufactured covers in the mechanical aspect, as there is no box to hold these harmonic covers. In this way, the aim is to analyse the behaviours of the different designs with respect to the printing directions, in order to be able to determine the relevance of this in their vibration [50].

**Figure 4:**
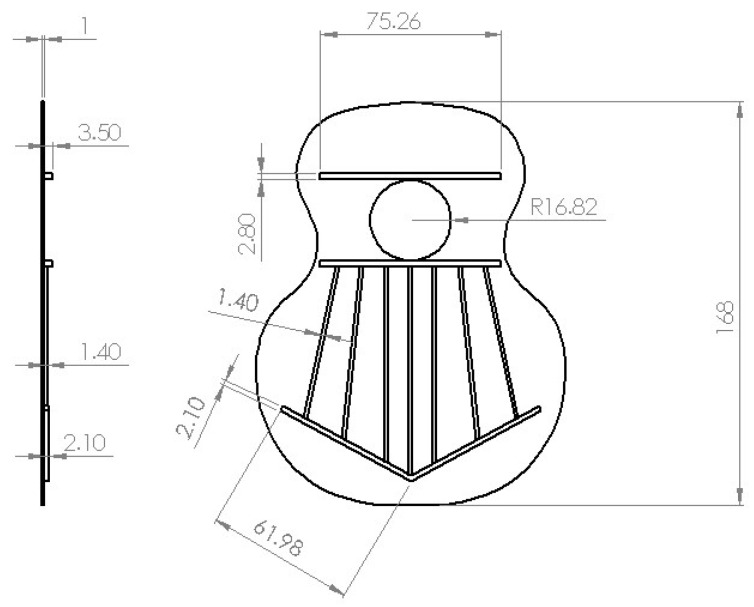
Top plate dimensions.

The dimensions used to carry out the different tests are shown in Figure 4, where the maximum dimensions of length and thickness of the covers are observed. Furthermore, the maximum height of the saddle in all the designs. The scale of reduction used with respect to an acoustic guitar at 1:1 scale was 0.28.

This reduction ratio favours the production of numerous models with the intention of observing the different behaviours of the models. Two tops of each type were also printed in order to observe the behaviour and quality of impression in the use of this type of manufacturing for the realisation of numerous tests, as shown in this work.

### 2.3. Vibration Analysis

As mentioned above, the main objective of this study was to analyse the first five vibrational modes selected for the study of the different designs of top plates created by AM, according to a previous study [44].

The vibration modes were initially tested by means of experimental tests, and then the tests were analysed by means of simulation. In this way, although the simulation did not include the manufacturing parameters, it was possible to compare the real results with those obtained virtually and, thus, to observe the reliability and predictive capacity of the behaviours of the harmonic caps and, subsequently, carry out an analysis of the repercussion of the manufacturing direction on the vibration behaviour.

#### 2.3.1. Analysis of Vibration Result: Physical Test

For the creation of the test, the experimental tests created by Chaldni [51] were taken into account. In these tests, Chaldni placed a plate on which he made it vibrate to observe the different vibration patterns according to the different harmonic frequencies associated with its vibration modes. This experiment was extrapolated to the study of guitar top plates, in which the sides of the top were fixed and without movement, as occurs in the assembly of the top with the sides of the guitar.

In addition, the driver was placed in the area of the lower ring where the bridge is located, thus simulating the behaviour of the bridge when the strings of the instrument vibrate. The bridge is the main area of vibration transmission and, therefore, of sound generation.

**Figure 5:**
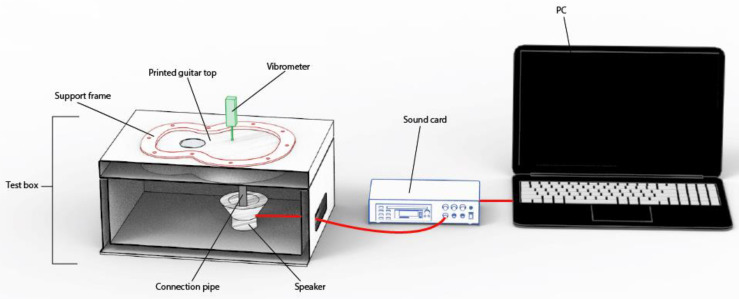
Outline of the experimental trial.

Once all the top plates were printed, they were placed individually on a structure created for the test, as shown in Figure 5. The physical test was carried out in a fully equipped engineering laboratory. The created covers had a reduced scale (Figure 3). In this way, a large number of different specimens can be studied. The printed specimens were not subjected to post-processing; therefore, the tested specimens presented the finish obtained from the print. In this study, the enclosure of the test structure is not relevant to the vibration analysis of the top plates, as the acoustics were not analysed, since what was carried out in this work was the exclusively vibrational analysis of the different top plates. Due to the large number of top plates to be tested, a continuous testing methodology was used. This consisted of printing the test specimens and then vibratory testing them instantaneously once printed, thus preventing the material used from ageing and deteriorating. Once the different top plates were tested, they were stored individually in sealed envelopes in order to conserve the properties of the caps in the best possible way.

The tests on each of the test specimens were carried out once and the results obtained between the two printed top plates were finally compared. It was observed that the vibrational response between both test pieces was similar, showing the reproducibility of the printer used for the experiments. This similarity in behaviour in results was observed in all types of printed and tested top plates.

The decision in this work not to carry out a structural study of the tops was due to the importance of defining the size, geometry and arrangement of the bridge that would be attached to each top. This variable is highly relevant, since the bridge serves as the link between the strings and the top, and the transmitter of the vibration when the strings are plucked. The transmission of the vibration and, therefore, the final sound of the instrument are affected by the bridge as well, as are variations in the design of the reinforcement structure of the top plate, as shown in a previous work [18].

Therefore, in order to test the mechanical integrity of the covers, it is necessary to analyse the vibration behaviour according to the reinforcement structures of each cover and, in this way, to define the geometry and position of the bridge with which the mechanical testing of the different specimens can be carried out.

The purpose of this structure is to hold the lid on its sides, preventing the lateral movement of the lid when it is excited by the loudspeaker. The design of the structure was based on an experimental design, as shown in Figure 4. The structure was made of wood, and the frame to hold the lids was printed in PLA material; the wood used was medium density fibreboard (MDF), which was selected as it is sufficiently thick to prevent vibration in the structure, thus increasing the reliability of the test. The cavity was designed to contain the driver. Finally, the loudspeaker structure was fastened with Velcro and screws for correct positioning to avoid loudspeaker vibration.

Each clamped top plate was in contact with the speaker at the bottom of the specimen by means of a plastic cylinder in contact with the top plate underneath it, so that the vibration patterns were clearly visualised on the front surface of the top plate at the moment of excitation. After placing all the elements shown in Figure 5, the salt was sprinkled on the test specimen using a sieve to avoid contact with the hands, and a frequency sweep was performed.

A Steinberg UR44 sound card was used to trigger the test, which was connected to the audio output of the PC, and the frequencies selected for the test were generated in the NCH Tone Generator software Version 7.05, obtaining control over the frequency sweep. Experimental data were collected in an Excel spreadsheet.

**Figure 6:**
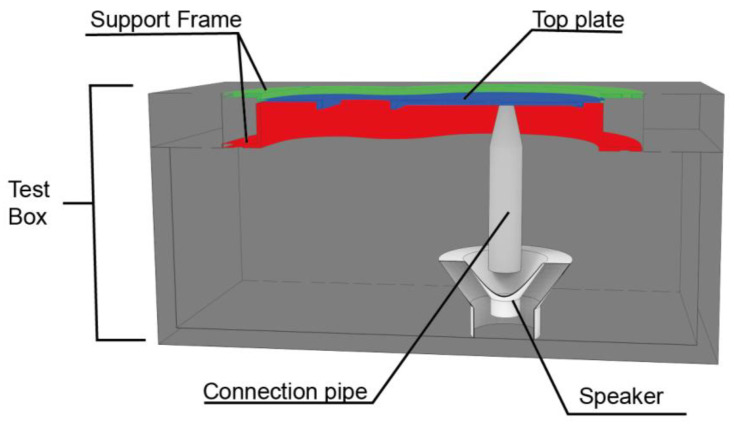
Detail of the fastening and generation of vibration on the top plate.

The frequencies related to the musical notes were not used, as the size of the top plate was very small and, therefore, the relationship of the frequencies associated with the musical notes in this case would not have been valid. Figure 5 and Figure 6 show the methodology used in the experimental test.

#### 2.3.2. Analysis of Vibration Result: Test Simulation by FEA

**Figure 7:**
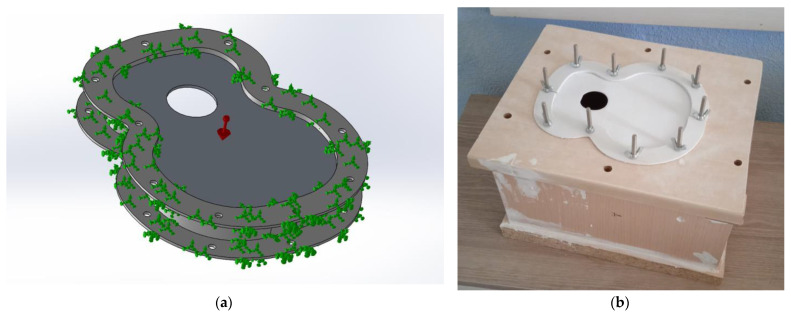
Subjects in the simulated test: (**a**) simulated model, in green the fixed connections are shown, and in red the gravity is shown.; (**b**) model placed for the experimental test.

The simulation of the vibration response of each of the designed covers was evaluated by FEA analysis in Solidworks, covering the first five vibration modes, as described in the physical experiment (Figure 7a). To compare the virtual results with the experimental results, a material with the characteristics of PLA used for the creation of the test pieces was created.

The use of simulation is important in physical tests in order to check whether the methodology used really provides a prior visualisation of the behaviour, so that customised designs can be made. Furthermore, in future studies, accelerometers can be used to analyse the points with the greatest vibration according to the vibration patterns previously obtained, with the intention of obtaining results of their harmonic frequencies.

In this work, the simulation of the behaviour with respect to withstanding the stresses caused by the guitar strings was not contemplated, as the study was based on analysing behaviour and reliability in terms of the visualisation of vibratory patterns. If this methodology is carried out with larger-scale tops, a stress study will be necessary to corroborate the behaviour of the material.

The study was limited to the five vibratory modes studied in the previous test, in order to obtain a visualisation of the Chladni figures associated with each of the frequencies obtained in the analysis [52,53].

When creating the test in the software, both the different printed top plates and the system for holding them were modelled. To accomplish this, the frames that pressed and held the test specimens were created. For this purpose, the clamped areas were created by replicating as closely as possible those used in the experimental test. Therefore, the joints between the two frames and the pressure zone on the lid were left fixed.

## 3. Results and Discussion

### 3.1. Results for Caps T01_90 and T02_90 Vibration Modes

**Figure 8:**
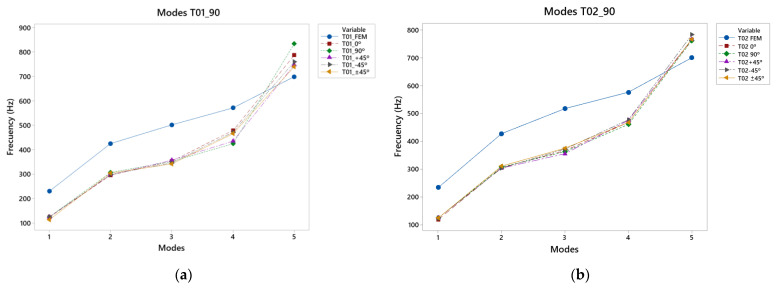
Design with neutral placement, 90° angle: (**a**) vibration modes T01_90, (**b**) vibration modes T02_90.

After the different tests, both experimental and virtual, the results obtained with angle 1 of 90° in the two designs, T01 and T02, show that in the FEM simulation, the frequencies obtained in the design T02_90 were lower than those in T01_90. In the fifth vibration mode with the T02 design, the results obtained were 701 Hz compared with 834 Hz for T01, as shown in Figure 8. This may have been caused by the guitar-bracing arrangement, as T01_90 uses a classical arrangement, based on Antonio de Torres’ design. This design means that there is not excessive reinforcement in the area of the major lobe and, therefore, the frequencies in the fifth mode are increased. On the other hand, the T02_90, which has greater reinforcement in the central area of the lower bout, reduces this response. In addition, the 90° angle causes a concentration of vibration in the bridge area.

In general, the results of the physical tests of both cover designs were lower than those of the simulation in the first four vibration modes. In the Appendix A, Table A1 and Table A2 show the vibration patterns obtained in the simulation and experimental tests of the T01_90 and T02_90 specimens, respectively, while the vibration response of the manufactured specimens is shown in the last specimen. In these experimental results, the greatest difference between both covers was found in the last two modes, where in T01, the covers printed in the 90° direction were higher than the other directions, obtaining 479 Hz. The direction of printing of −45° in T02 had a higher frequency than the other directions, which means that for frequencies in the range of 400–500 Hz, the top plates with a direction that reinforces the lower bout and the bass area had a better response for high frequencies.

In the fifth vibration mode, the greatest differences between printing directions were observed, as in T01, the frequencies reached were higher than those in the virtual results, and the 180° direction reached 834 Hz, the highest frequency obtained by both types of specimen, whose angle provided a greater vibration resembling the wooden top plates, causing this vibration to be transmitted from the lower bout to the upper bout. On the other hand, in T02, the tendency of the −45° directions continued, which may have been caused by the greater reinforcement in the bridge area due to the distribution of the bracing, so that the bass area was more important as the lower bout was reinforced. With respect to the simulation, in both cases, the frequencies of the first to the fourth mode were below those of the simulation, whereas in the last mode, where the frequencies were higher, the frequencies of both were higher, so the use of PLA and such bracing designs provided a greater response at high frequencies.

With respect to the approximate percentages of the error between the results of the simulation and the experimental tests, in the case of the T01_90 and T02_90, the first vibratory mode was where the greatest difference in results was shown, with an error of 85%; with respect to the next two vibratory modes the error was 40%, the fourth mode was at a percentage of 24% and, finally, the last mode with the highest frequency had a percentage of 11% or lower. These percentages were applied to each of the different top plates in their printing direction. In conclusion, the error rate decreased with increasing frequencies.

### 3.2. Results of Caps T01_81 and T02_81 Vibration Modes

**Figure 9:**
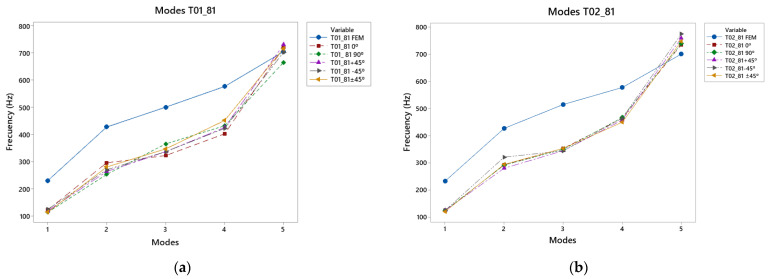
81° angle design: (**a**) vibration modes T01_81, (**b**) vibration modes T02_81.

The results for the T01_81 and T02_81 top plates are shown in Figure 9 and Table A3, respectively, and in Table A4. It can be seen that the first four modes of the T01_81 had higher frequencies compared to the frequencies of the T02_81, caused by the reinforcement of the bracing, as well as the T02; however, in the last mode, where the frequencies were higher, the T02_81 reached higher frequencies, more specifically those that were manufactured in the −45° direction. This was related to the lower reinforcement in the bass area, compensating for the reinforcement in the treble area by the diagonal lower harmonic woofer and woofer. In the frequency range of 300–450 Hz, the 90° direction showed a higher frequency in T01_81, whereas in the latter mode, the +45° direction caused a good response at frequencies from 700 Hz upwards. Compared to T02_81, the −45° direction presented higher frequencies up to 775 Hz, which was caused by the reinforcement of the top plate in the center and its tendency to vibrate in the bass area, compensating for the reinforcement produced in the treble area.

With respect to the simulation, the experimental results were lower than those in the simulation, except in the last mode, where the frequencies were higher, showing the effect of the manufacture on the internal structure of the material affects.

In this case, the difference in the error between both measurements of the two top plates, T011_81 and T02_81, show greater differences between them, since in the first mode, their error difference was 84%; the difference between the following two modes was 45%, displaying a greater difference in comparison with the top plates of the previous point. In the fourth mode, the T01_81 caps had a relative error of 35%, while the T02_81 caps did not exceed 27%, a very important aspect for high frequency ranges, as shown in the fifth mode, where the error in T01_81 did not exceed 5%, while in T02_81, it reached 10%.

### 3.3. Results of Caps T01_99 and T02_99 Vibration Modes

**Figure 10:**
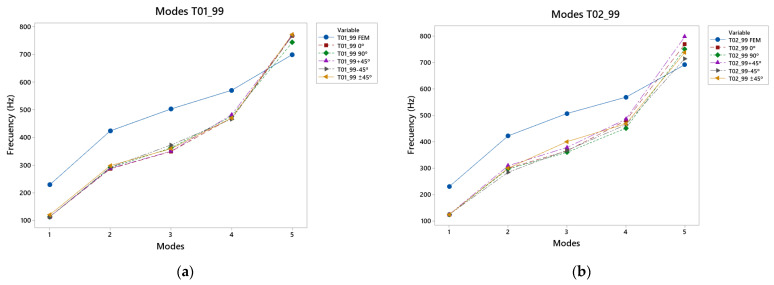
99° angle design: (**a**) vibration modes T01_99, (**b**) vibration modes T02_99.

Figure 10 shows the results of the vibrational modes for the T01_99 and T02_99 top plates, and Table A5 and Table A6. In this case, the diagonal lower harmonic variate was reinforced by the bass area. In mode 1, all the printed caps had similar frequencies, but were lower than the results in the simulation. The top plates presented similar frequencies, but these were lower than those in the simulation. This result occurred in the second vibration mode, in which the vibrations were concentrated in the larger lobe. As the frequency increased, in mode 3, it was observed that the stronger reinforcement in the treble area did not provide good displacement, but in the −45° direction, together with the reinforcement of the fan in this area. In the fourth vibratory mode, the +45° direction covers became more important. This was due to the fact that as the frequencies increased and the notes were higher, this angle caused the vibration to be directed to the correct zone, which is why these covers presented higher displacements in comparison with the others.

Finally, in the fifth mode, where the greatest differences between the two types of top plate were observed, it should be noted that the T02_99 reached higher frequencies with the angle of +45°, so that this direction of manufacture as the frequencies were raised meant that the vibration was favored at higher frequencies. It should also be noted that this was the only vibration mode in which the experimental results exceeded the virtually obtained results.

With respect to the differences between the results of both the T01_99 and the T02_99 top plates, in the first mode, the difference did not exceed 85%; in the following two modes, it did not exceed 40%, as with the previously analysed T01_90 and T02_90 top plates. In the last mode, with the highest frequencies, 11% was not exceeded, but this was not as low as in the previous case of top plates.

### 3.4. Overall Results according to the Direction of Printing

After analysing the different specimens tested and comparing the experimental and virtual results, we proceeded with an analysis that focused on the effects of the printing direction on the vibration results of the manufactured specimens.

**Figure 11:**
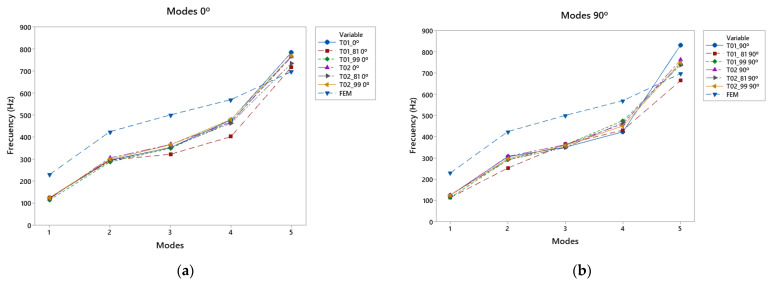
Comparative modal response as a function of print angle: (**a**) vibration modes 0°, (**b**) vibration modes 90°.

Figure 11a shows a comparison of the different specimens with the 0° direction. In this case, the response in the fifth mode of all the specimens was higher than that of the simulation, presenting a greater response at higher frequencies. On the other hand, in the other modes, the simulation was above the experimental trial. In this case, the T01_81 top plate showed the weakest behaviour when compared to the other test specimens. This may have been caused by stronger reinforcement in the thinner chord areas, leaving less reinforcement in the lower bout area at low frequencies, so that the vibrational response was not increased by a reduction in the reinforcement in the thicker chord area. This can be compared with T02_81, which had stronger reinforcement of the larger lobe and showed a better response at low frequencies.

In Figure 11b, for frequencies from 350 to 480 Hz, the 90° printing direction favored vibration in the third vibratory mode, which may have been caused by the transmission of the vibration of the lower bout to the lower region of the top plate. At low frequencies in the first two modes, the T01_81 still showed a weaker response in these modes because the low-frequency area of the top was not reinforced to a large extent. On the other hand, the top plates with more reinforcement in this area, as in the cases of T02_90 and T02_99, presented better responses due to the reinforcement of the bracing guide.

**Figure 12:**
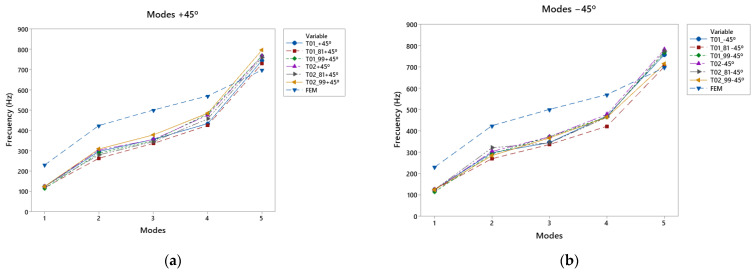
Comparative modal response as a function of print angle: (**a**) vibration modes +45°, (**b**) vibration modes −45°.

The +45° direction caused an increase in frequencies in the 450–840 Hz range of 11.6% with respect to the other directions (Figure 12a), except for the 90° direction, which achieved an increase in frequencies in the fifth mode of 16.5%. It is worth highlighting the strong response of the T02_99 in the fifth mode, with a greater reinforcement in the area of the lower bout, but that was compensated with this direction of impression. The reinforcement in the low-frequency region, together with the direction of the impression, generated a good response in all the modes studied.

The printed top plates in the −45° direction had a clear influence on the low frequencies (Figure 12b), presenting a good frequency response in the range of 0 to 125 Hz, producing a displacement of the vibration patterns towards this direction, and favoring the low frequencies, in comparison with the other printing directions.

**Figure 13:**
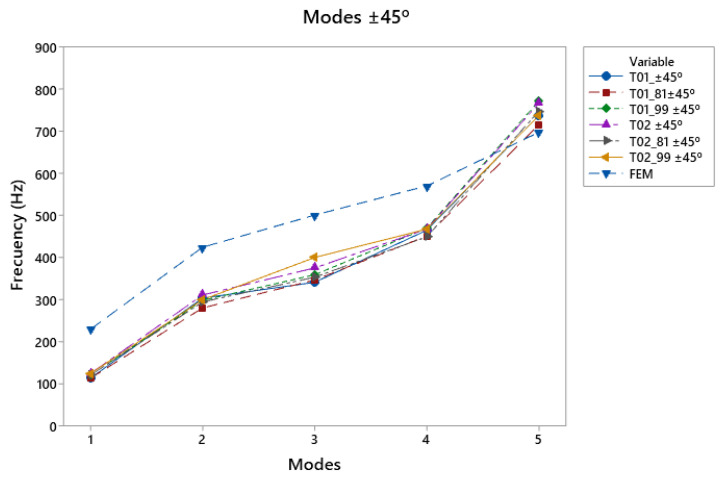
Comparative modal response as a function of print angle; Vibration modes ±45°.

The ±45° direction (Figure 13d), where the reinforcement of the manufacturing caused greater stiffness in the specimen, ensured that, in the first vibratory mode, where the vibration was concentrated in the lower bout, with respect to the other directions, the frequencies were 8.8% lower than the others. In this case, the T02_90 top plates showed a good response with a double reinforcement, which was generated by the direction of impression. It had a good response at high and low frequencies, and in the T02_99, thanks to the reinforcement of the structure in the low-frequency zone of the lower bout, the response in the third mode was good.

## 4. Conclusions

The methodology designed and specified in this work adds a new approach to the design and analysis of acoustic guitars. The use of FFF technology has been studied as a fundamental tool for the development and final design of custom guitars. Accordingly, the modification and creation of guitars by using this technology can be adapted to the tastes of the guitarist, since it is possible to parameterise the geometry and 3D printing properties to carry out a completely customised design. From the tests carried out and after analysing the different results obtained in this study, the following conclusions can be drawn:

The parameterisation of the guitar bracing generates different vibration patterns, depending on the frequency and, thus, the vibration of the tested top plates. The simulation ed a pattern that was very similar to the experimental patterns, which is very important as it provides an approximate idea of the vibration behaviour, showing the tendency of its movement.

One aspect to be improved in the virtual tests is the need to include the printing directions in the 3D models to increase the reliability of the simulation with respect to the experiment.

Considering the printing direction, if printed at an angle of 180°, the movement on the surface of the lid is greater because, as with wooden top plates, the vibrations are transmitted more easily in all its regions as it behaves like the grain of the wood. On the other hand, the 0° angle does not allow the vibration to pass from the lower bout to the upper bout easily, reducing the displacement in the lower bout and reducing its response at high and low frequencies. In order to enhance the movement in the zone of the first three strings (right zone), the reinforcement should not reinforce this zone excessively, as in the case of the T01_81 top plates, and the printing angle should also be +45°. In contrast to the T01_81 top plates, in order to obtain greater movement in the area of the last three strings, less reinforcement is required in the left area so as not to limit its movement; therefore, an angle of −45° favours the transmission of vibration in this area. The behaviour of the upper bout is influenced by the reinforcement of the top plate; if it is printed at ±45°, the lower bout hardly shows any movement, as it is mostly reinforced by the different printing lines and the presence of the guitar bracing, but the upper bout gives a good performance as it is the least reinforced area, since there is only one harmonic rod in this area, separating and reinforcing the upper bout from the other parts of the top plate.

Moreover, the use of desktop printers for the manufacture of the different top plates makes it easier to test numerous designs in a quick and easy way. In this sense, the increase in the size of the top plates and, therefore, in the instrument, increases, The different AM technologies available will be considered, as well as the material used, as studies can be carried out with different materials. A comparative analysis with a real wooden top is underway for a future publication, in which aspects of structural and acoustic aspects will be analysed, as this work started with a vibration analysis and the determination of the methodology used for future comparisons.

## Figures and Tables

**Table 1:** Vibrometer data Wintact WT63B.

Information	Data
Acceleration	0.1~199.9 m/s^2^
Speed	0.1~199.9 mm/s
Displacement	0.001~1.999 mm
Accuracy vibration displacement	0.01~0.02 mm, ≤ ±10%, ≥0.02 mm, ≤±5%
Vibration speed accuracy	0~2.0 mm/s, ≤ ±10%, ≥2.0 mm/s, ≤±5%
Accuracy acceleration of vibration	0~2.0 mm/s^2^, ≤ ±10%, ≥2.0 mm/s^2^, ≤±5%
High frequency	1 KHz~15 KHz (HI)
Low frequency	20 Hz~1 KHz (LO)

**Table 2:** Printing directions and coding of specimens.

Angles	Angles	Angles	Angles	Angles
0°	90°	+45°	−45°	±45°
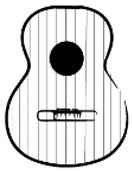	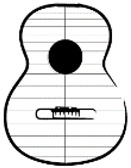	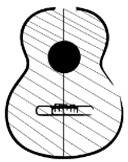	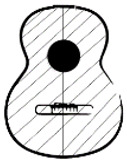	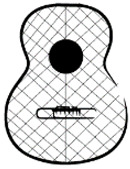

Due to the large number of top plates to be tested, a procedure was carried out.

## Data Availability

Data are contained within the article.

## Appendix A

**Table A1:** Vibration patterns T01_90.

	Modes	M1	M2	M3	M4	M5
	FEM					
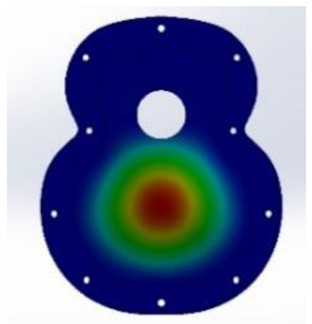
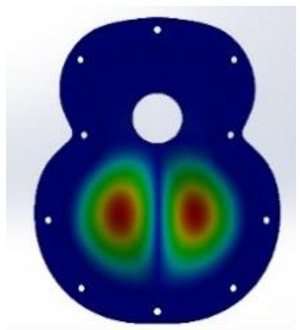
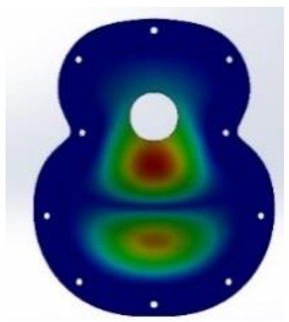
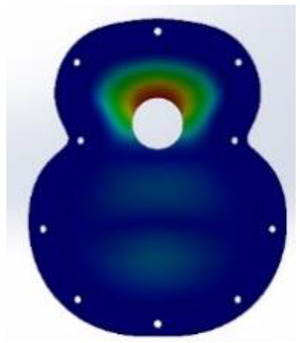
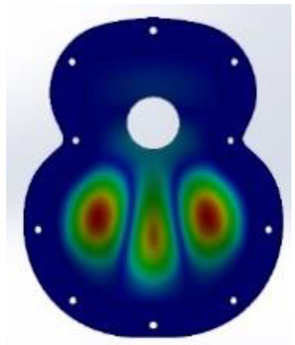
	T01_90 printing angle 0°					
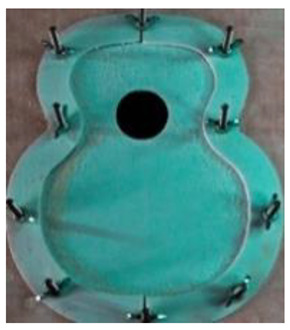
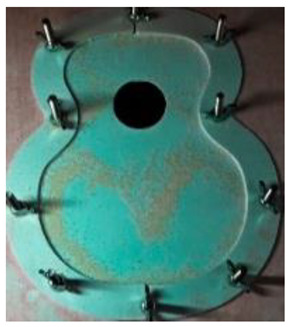
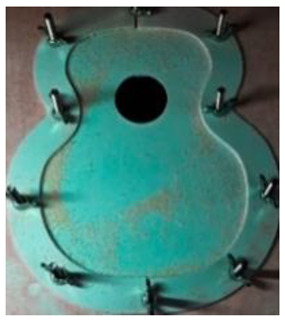
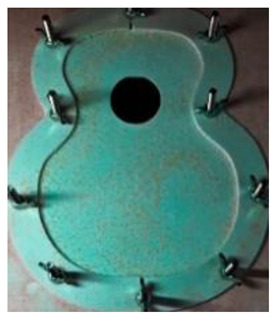
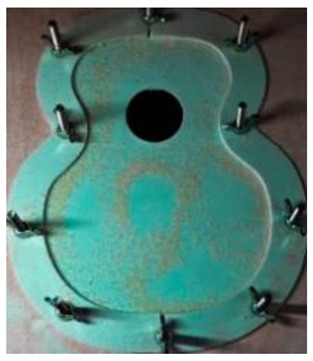
	T01_90 printing angle 0°					
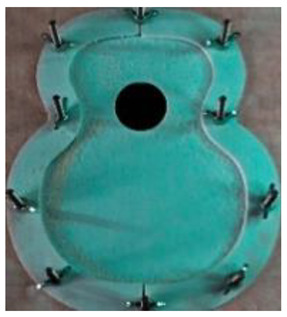
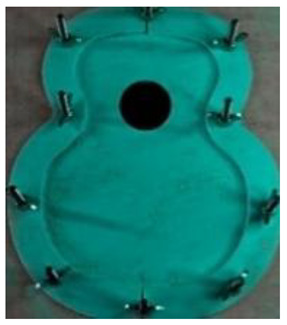
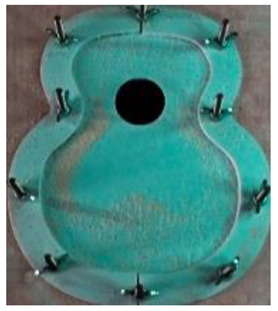
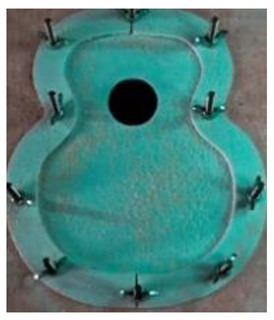
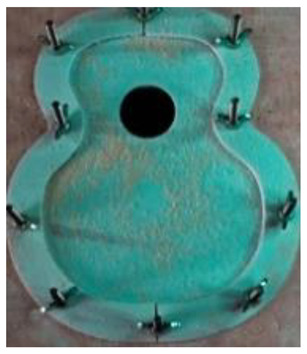
	T01_90 printing angle 90°					
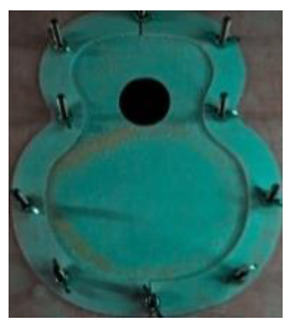
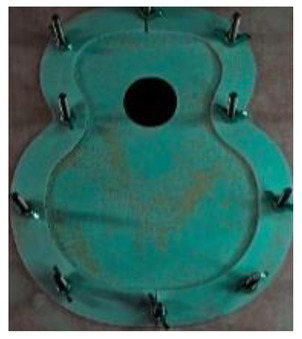
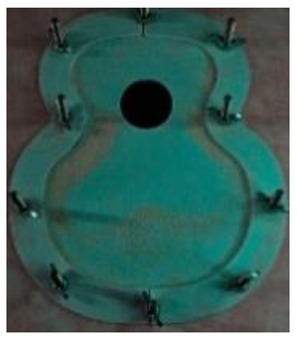
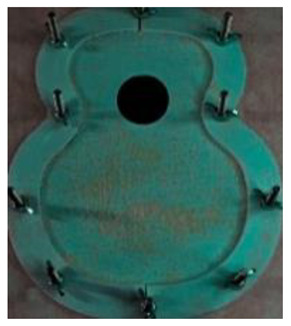
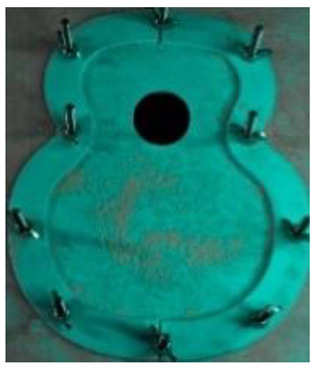
	T01_90 printing angle 90°					
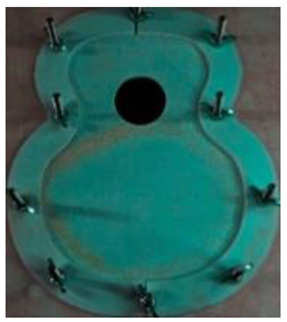
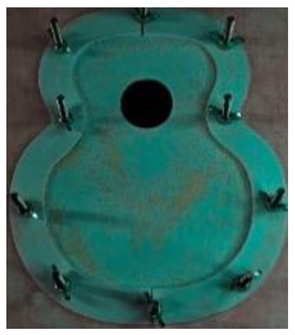
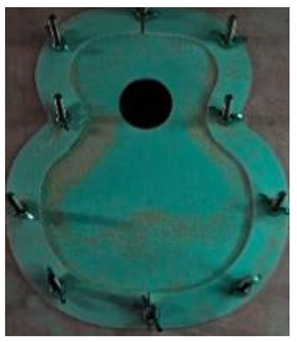
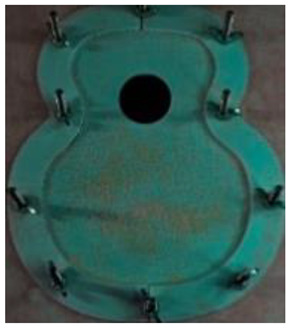
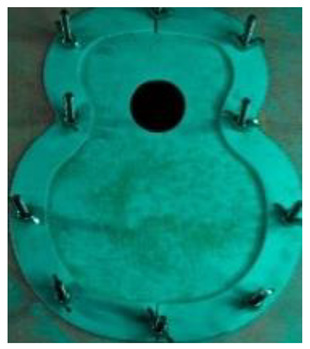
T01_90 printing angle +45°						
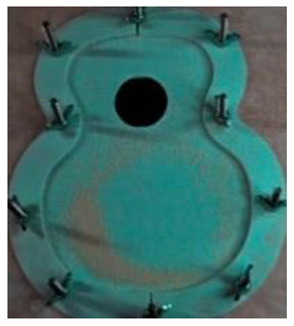
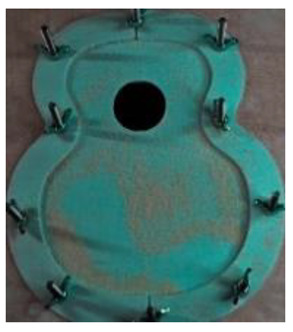
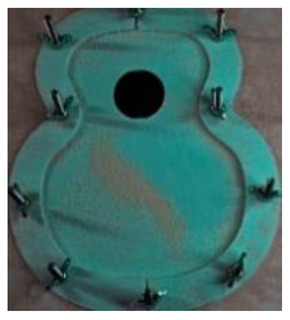
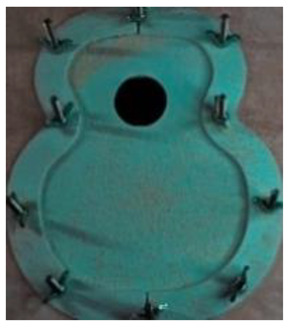
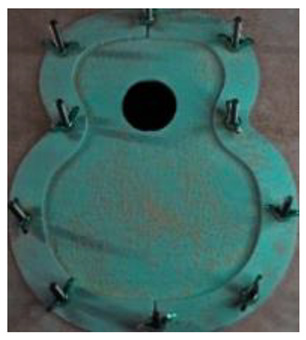
	T01_90 printing angle +45°					
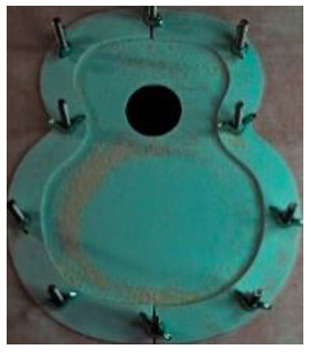
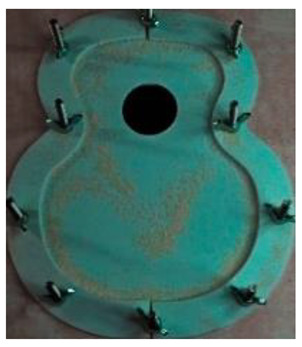
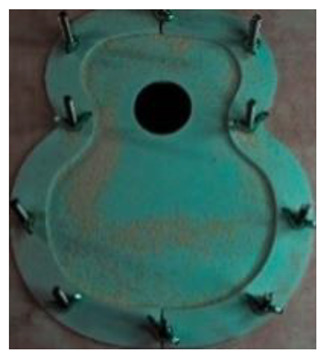
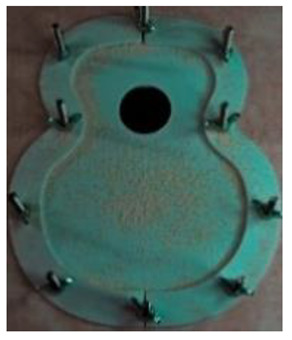
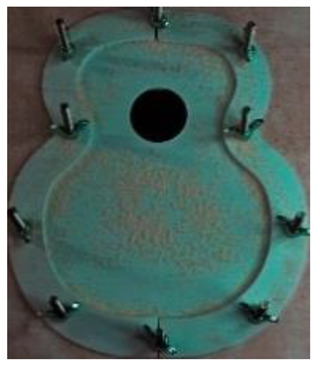
	T01_90 printing angle −45°					
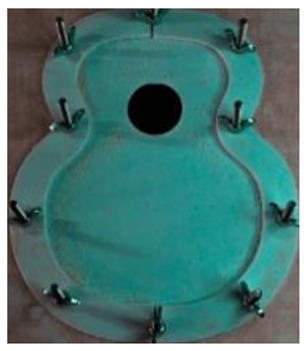
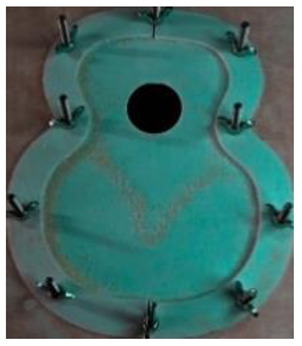
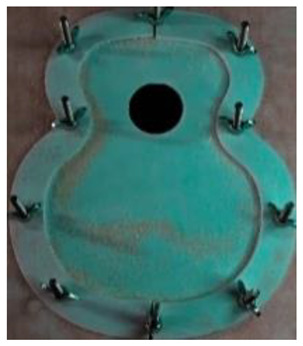
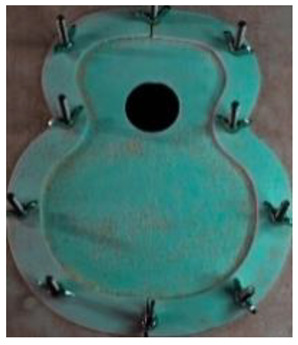
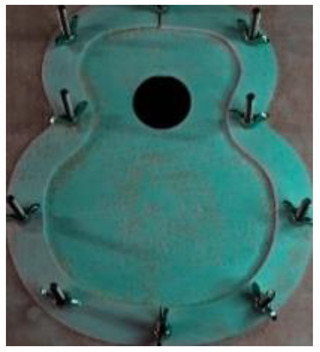
	T01_90 printing angle −45°					
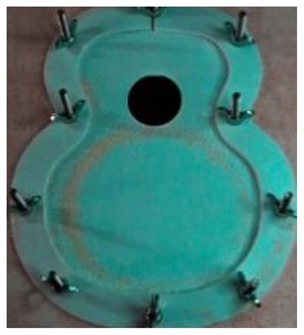
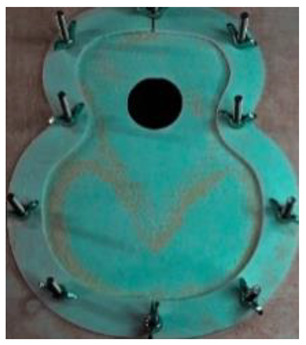
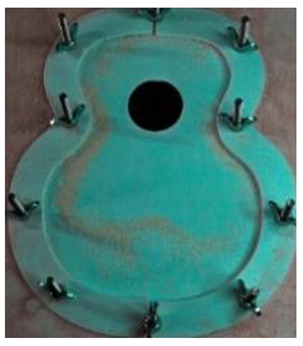
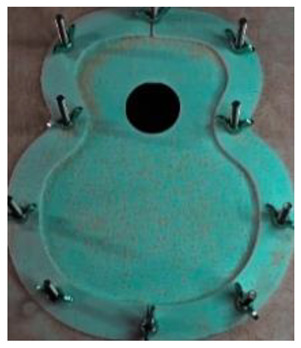
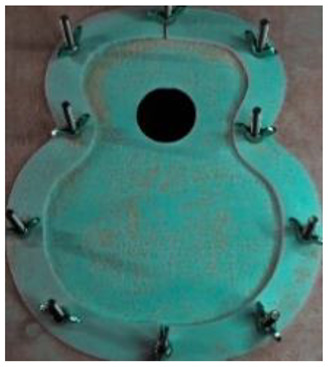
	T01_90 printing angle ±45°					
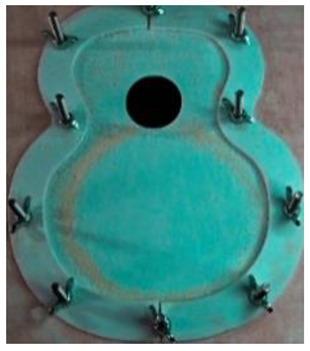
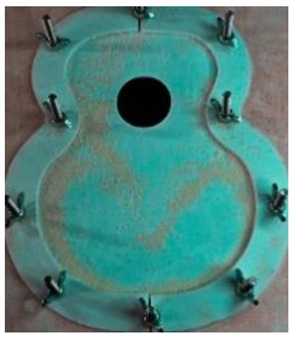
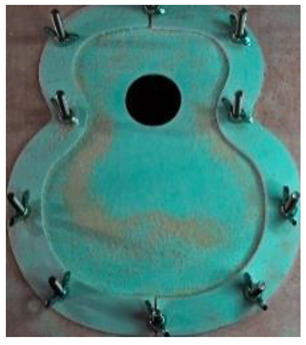
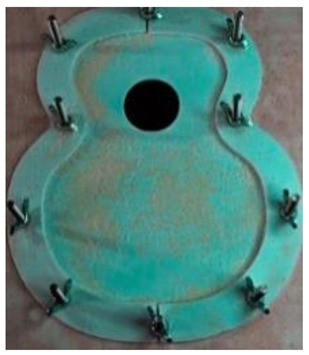
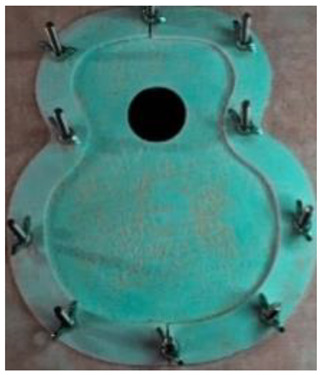
	T01_90 printing angle ±45°					
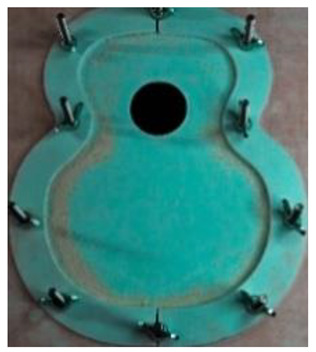
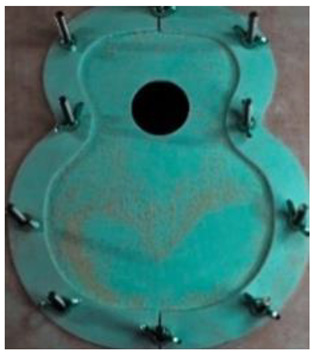
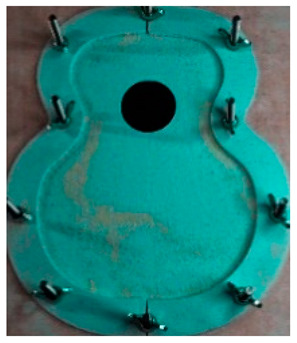
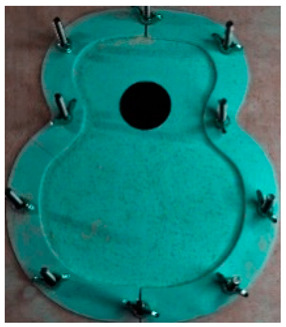
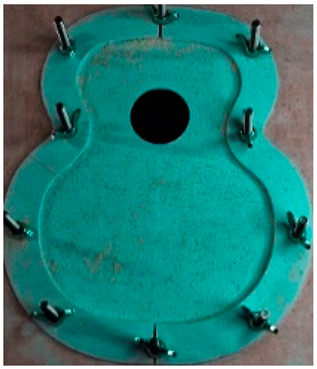

**Table A2:** Vibration patterns T02_90.

Modes	M1	M2	M3	M4	M5
FEM					
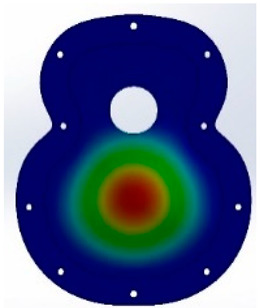
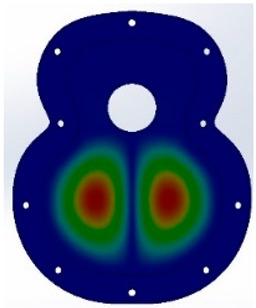
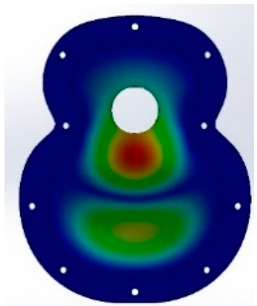
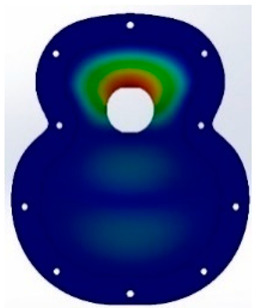
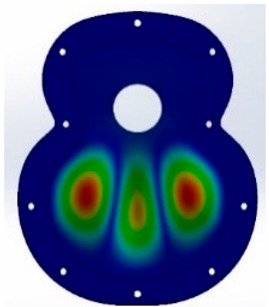
T02_90 printing angle 0°					
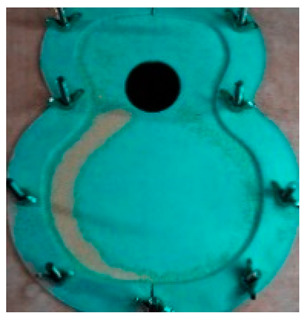
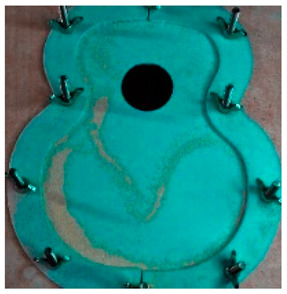
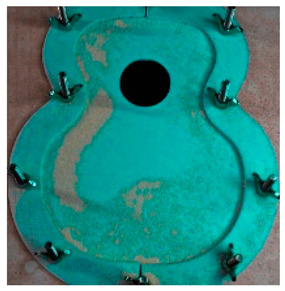
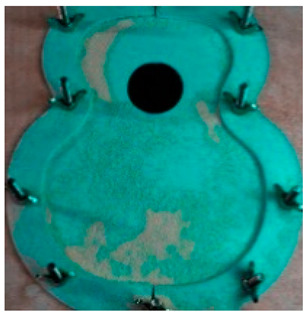
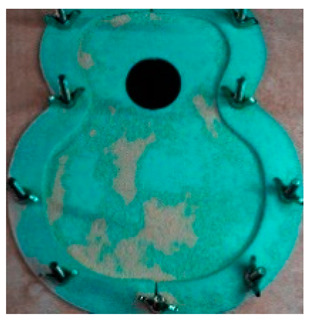
T02_90 printing angle 0°					
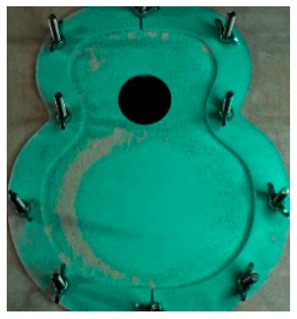
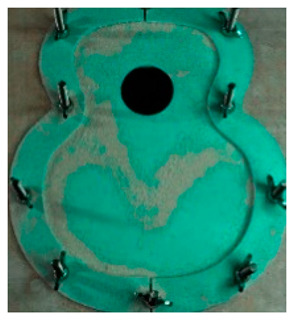
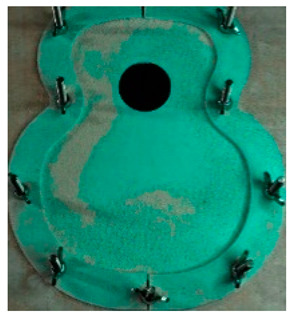
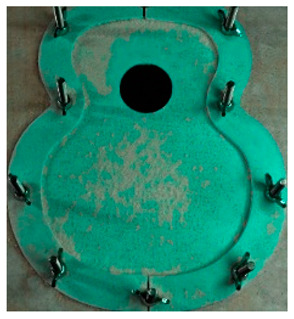
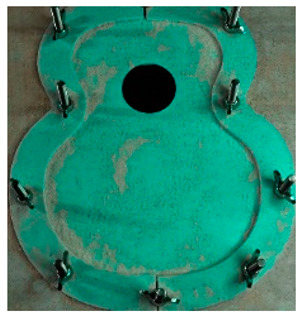
T02_90 printing angle 90°					
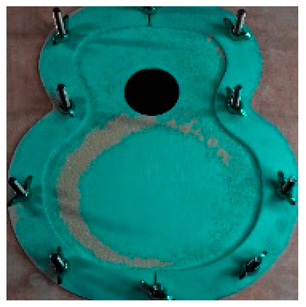
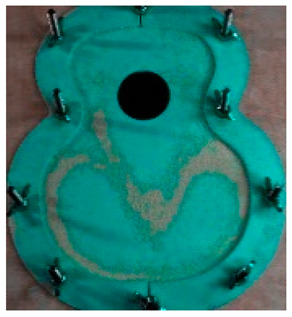
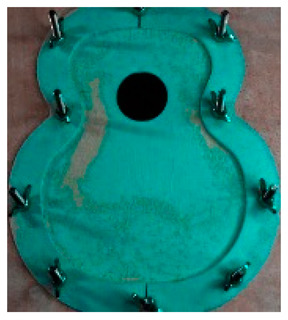
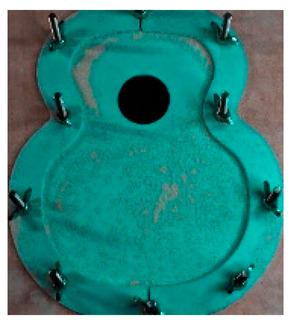
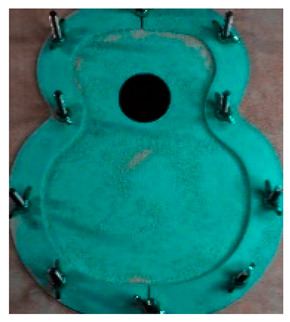
T02_90 printing angle 90°					
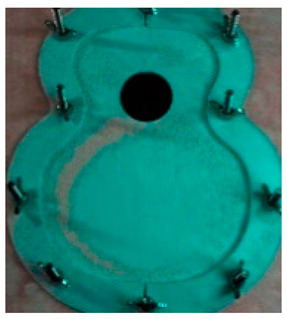
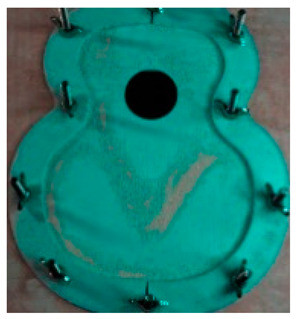
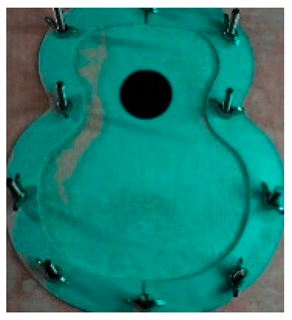
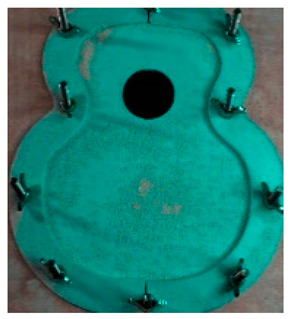
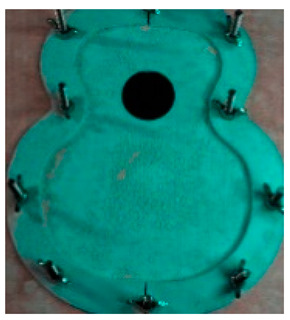
T02_90 printing angle +45°					
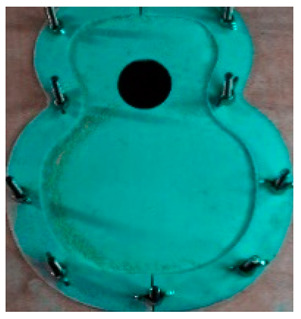
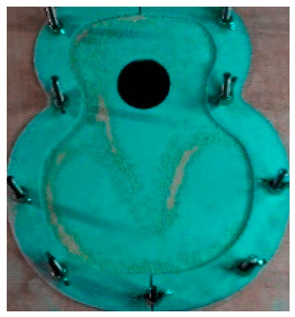
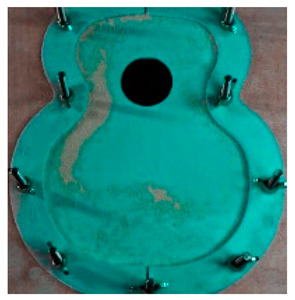
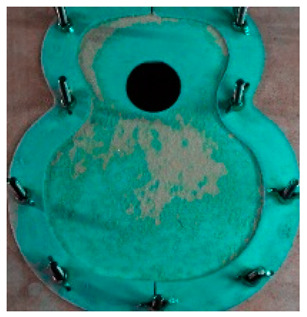
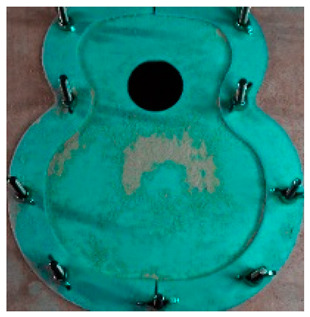
T02_90 printing angle +45°					
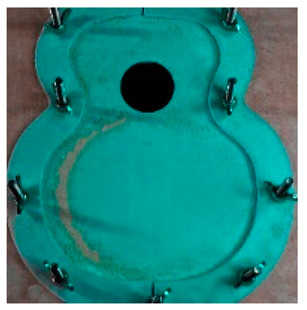
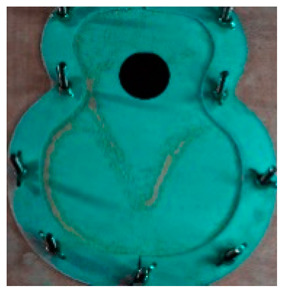
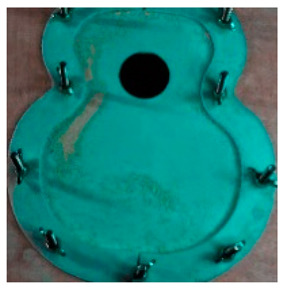
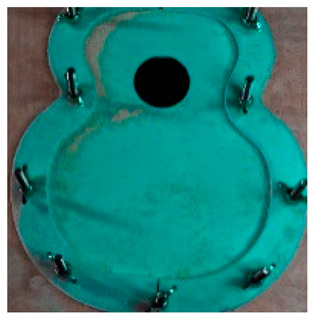
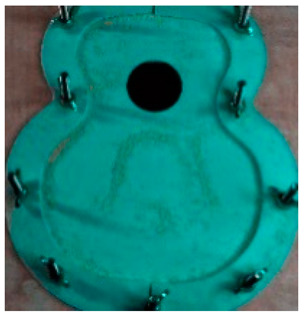
T02_90 printing angle −45°					
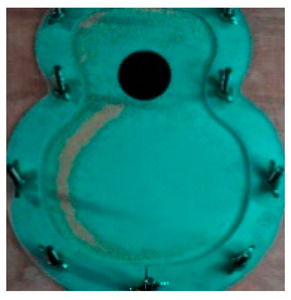
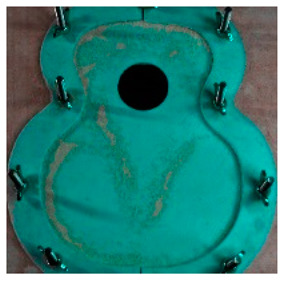
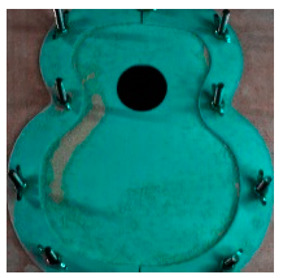
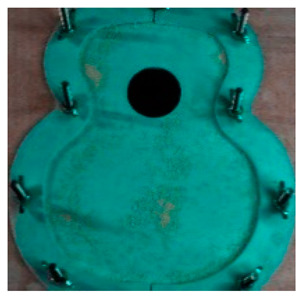
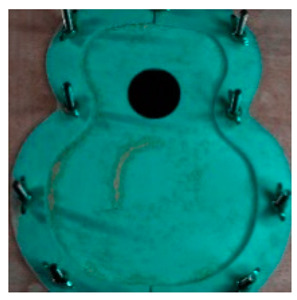
T02_90 printing angle −45°					
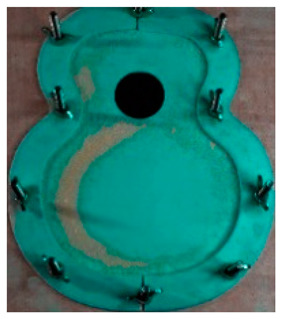
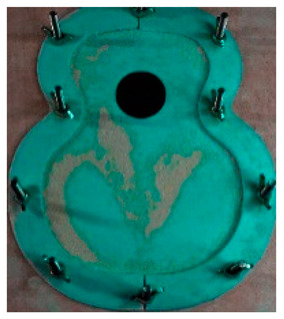
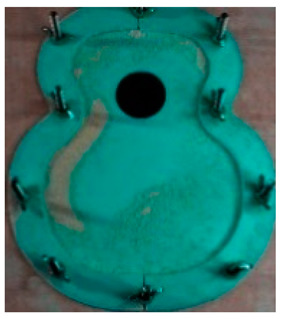
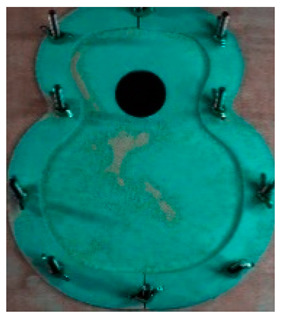
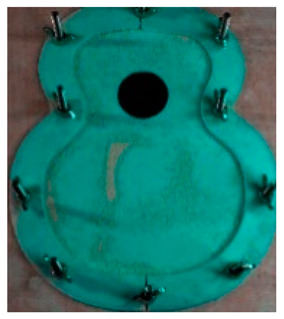
T02_90 printing angle ±45°					
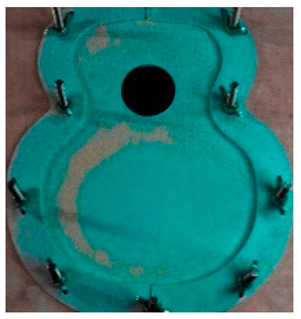
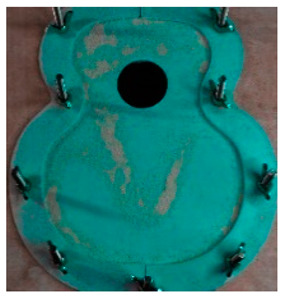
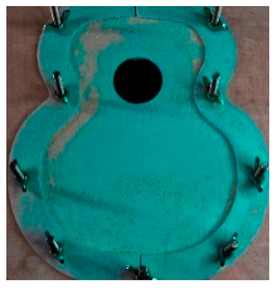
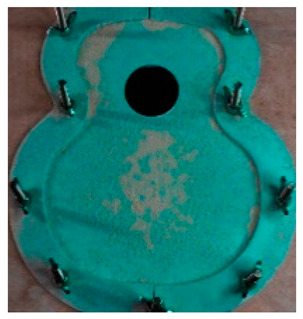
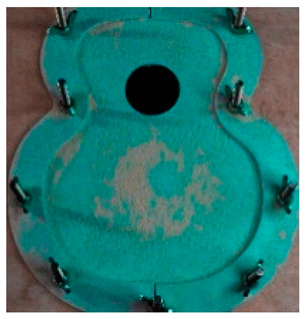
T02_90 printing angle ±45°					
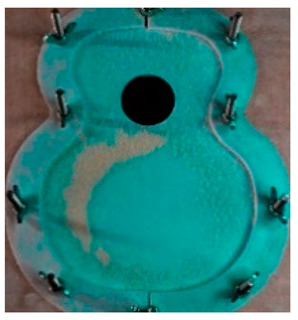
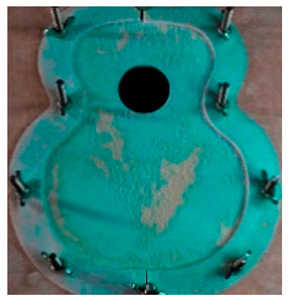
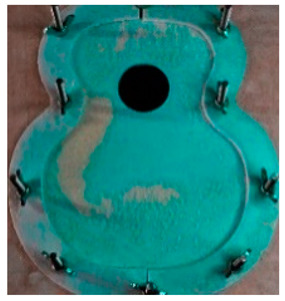
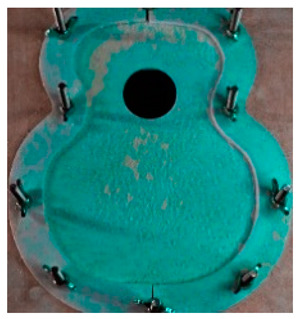
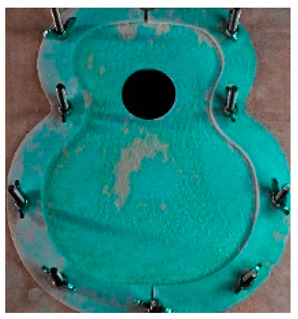

**Table A3:** Vibration patterns T01_81.

Modes	M1	M2	M3	M4	M5
FEM					
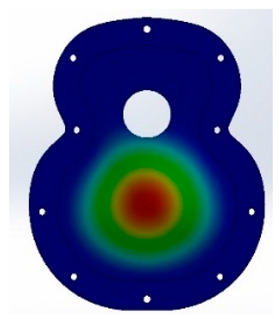
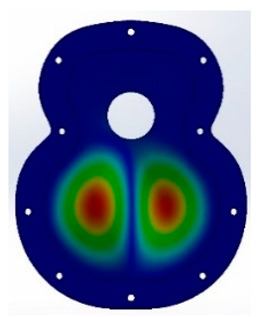
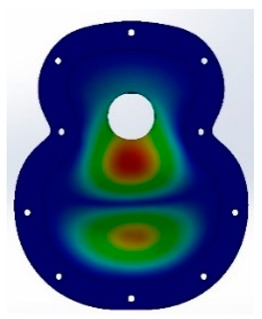
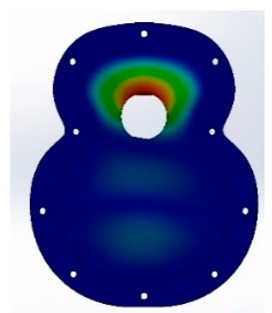
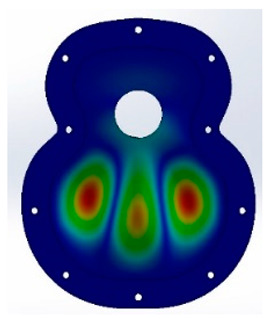
T01_81 printing angle 0°					
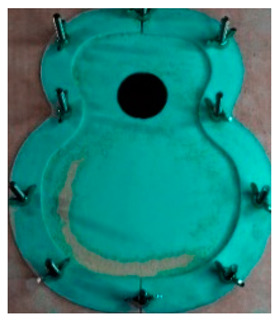
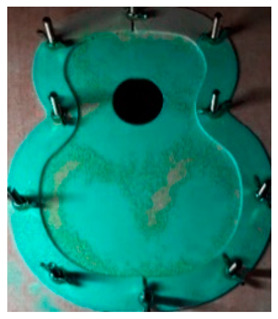
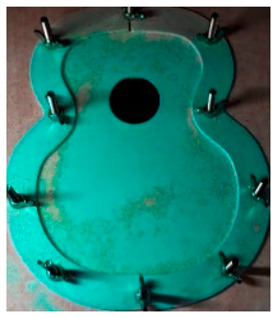
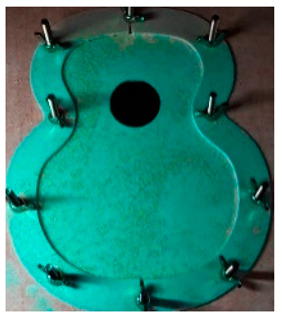
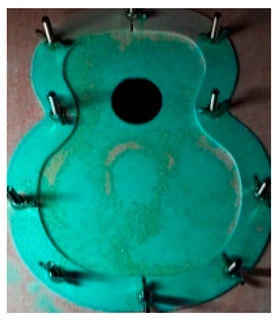
T01_81 printing angle 0°					
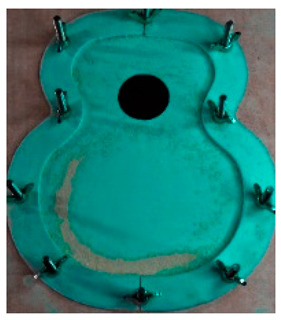
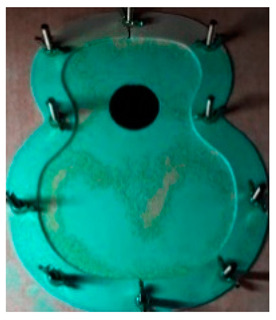
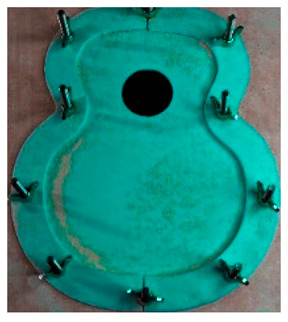
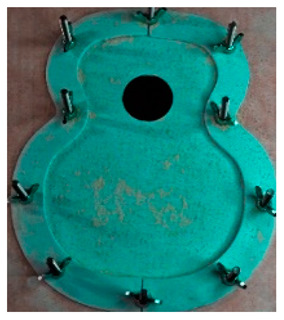
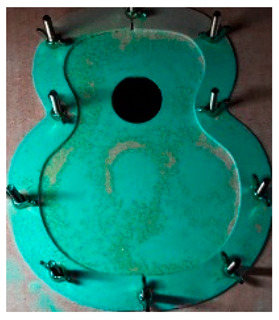
T01_81 printing angle 90°					
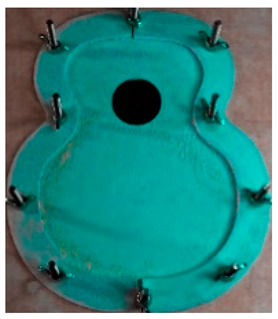
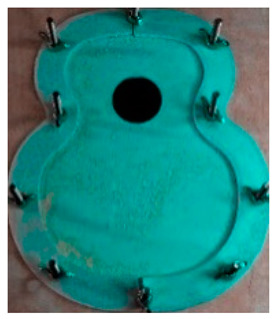
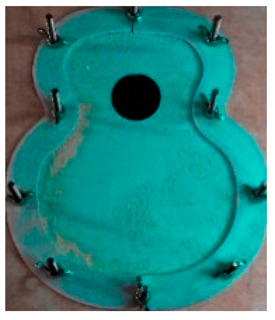
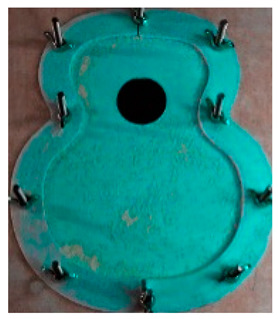
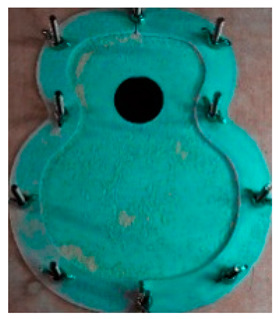
T01_81 printing angle 90°					
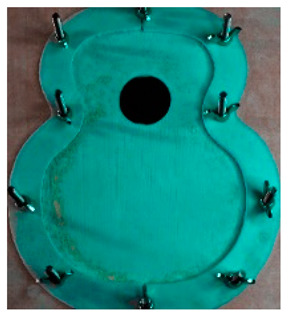
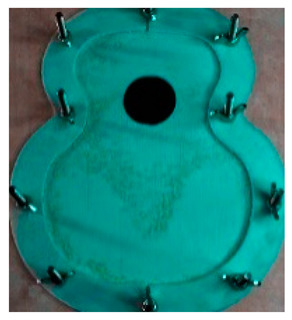
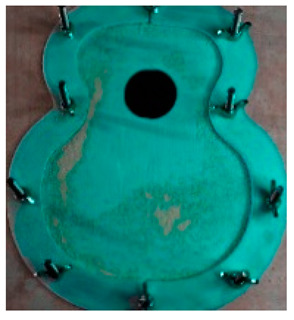
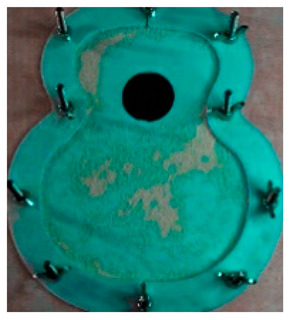
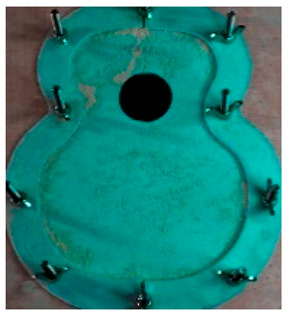
T01_81 printing angle +45°					
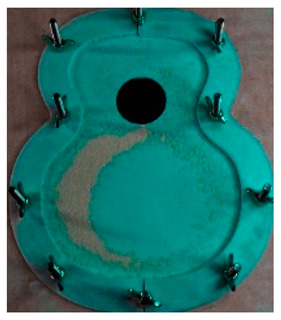
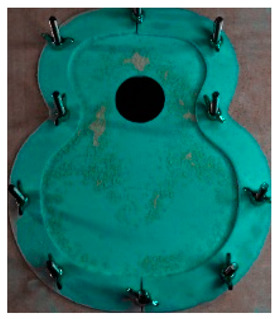
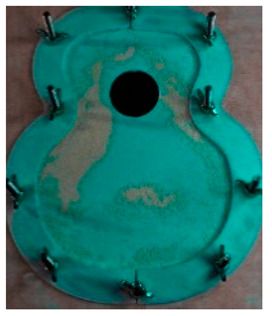
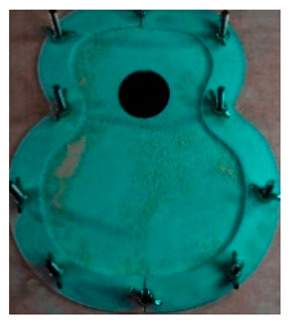
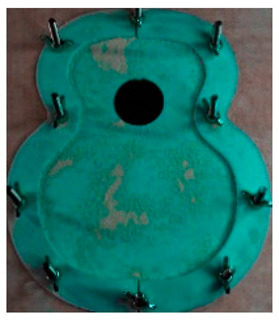
T01_81 printing angle +45°					
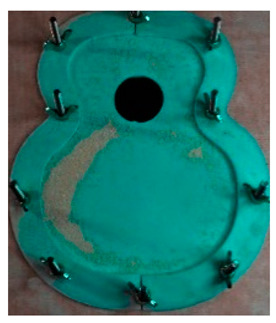
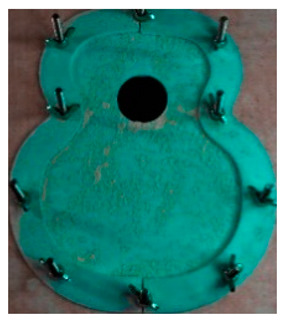
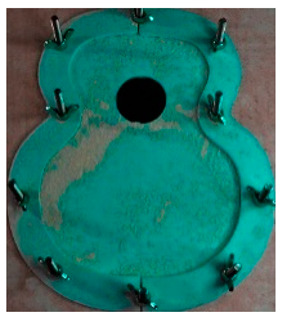
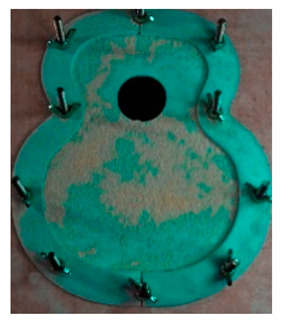
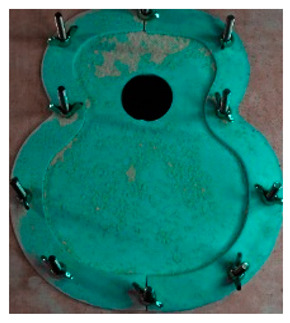
T01_81 printing angle −45°					
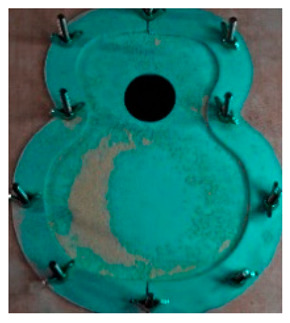
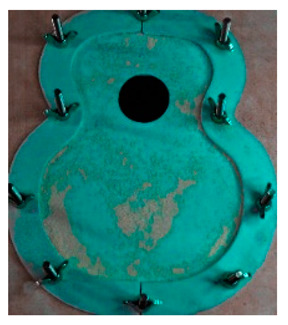
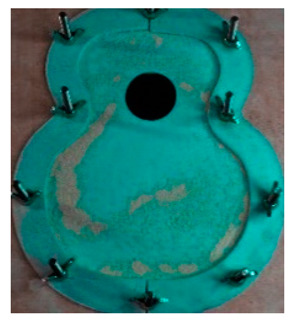
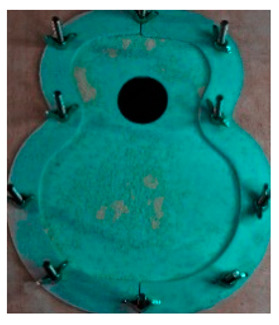
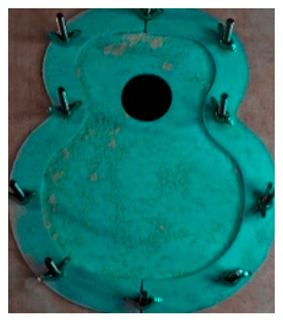
T01_81 printing angle −45°					
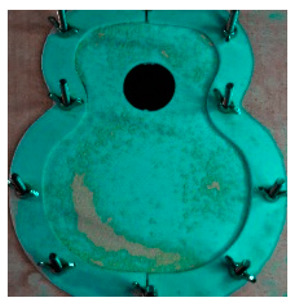
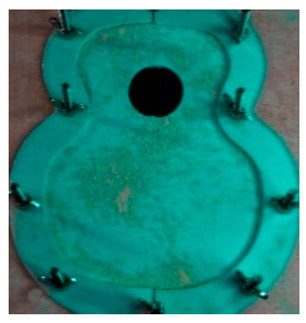
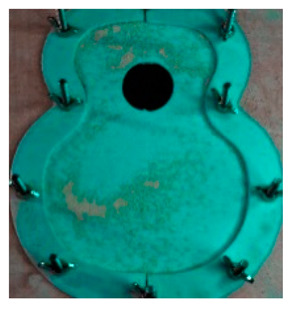
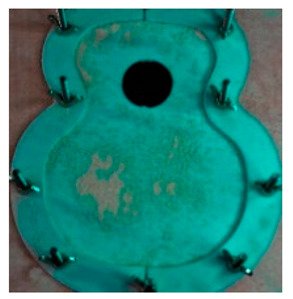
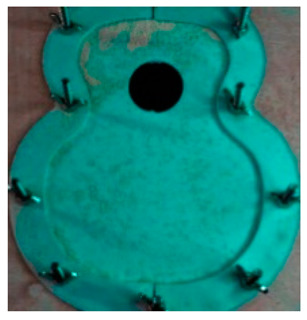
T01_81 printing angle ±45°					
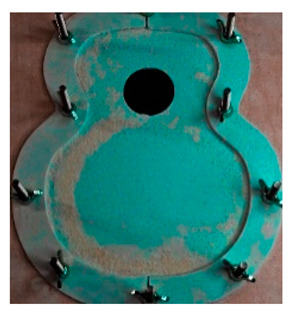
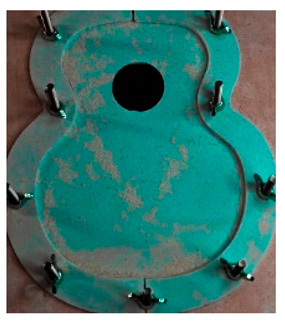
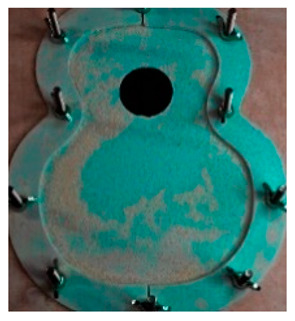
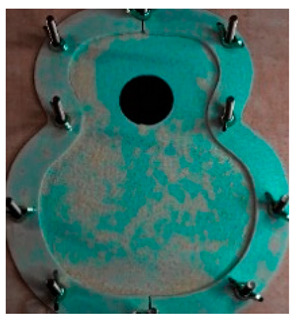
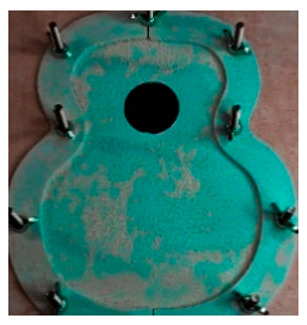
T01_81 printing angle ±45°					
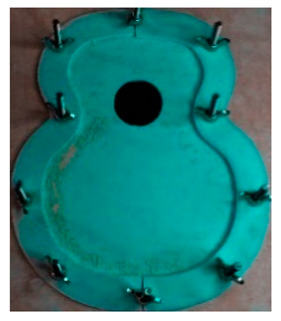
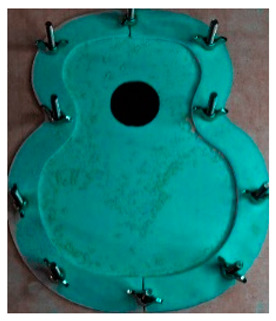
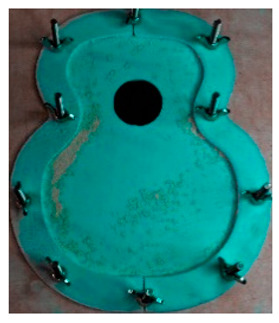
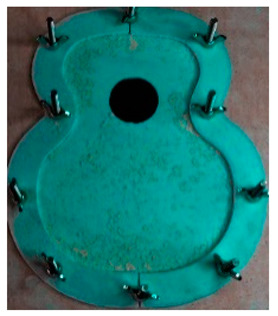
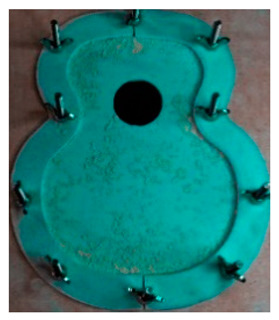

**Table A4:** Vibration patterns T02_81.

Modes	M1	M2	M3	M4	M5
FEM					
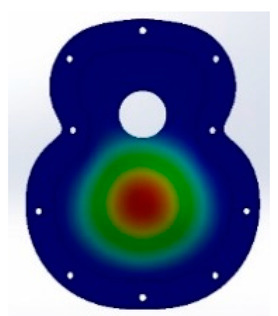
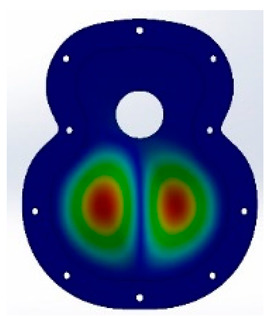
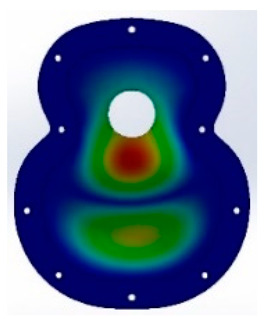
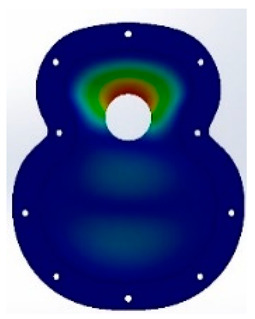
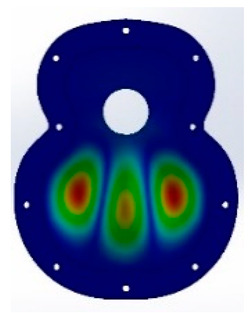
T02_81 printing angle 0°					
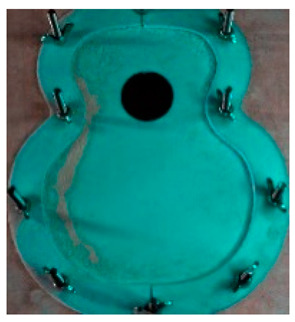
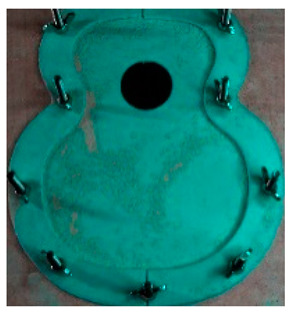
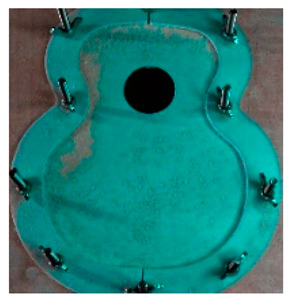
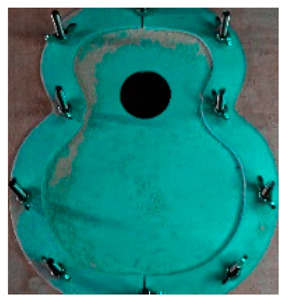
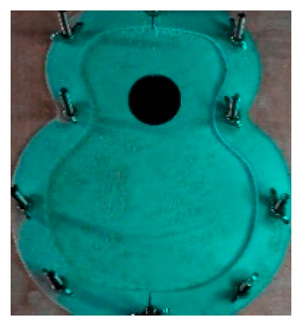
T02_81 printing angle 0°					
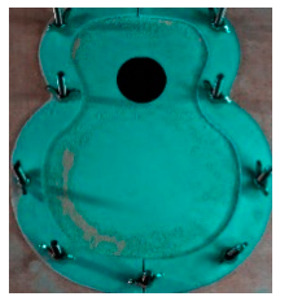
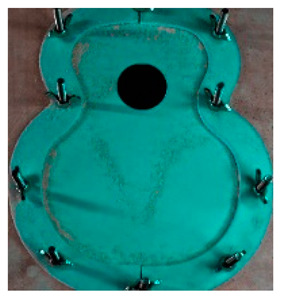
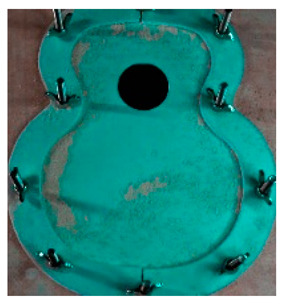
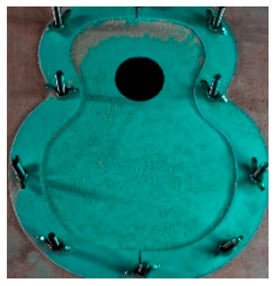
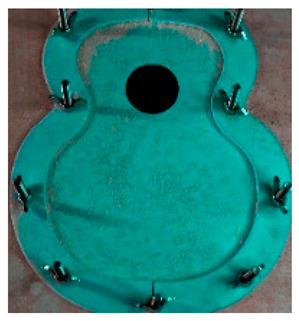
T02_81 printing angle 90°					
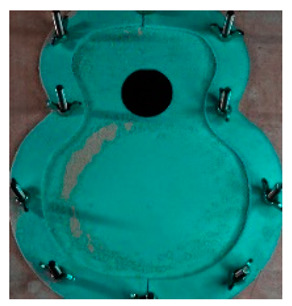
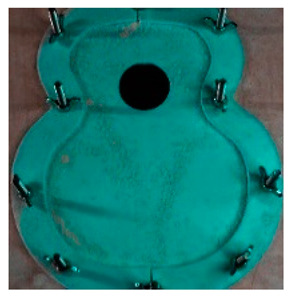
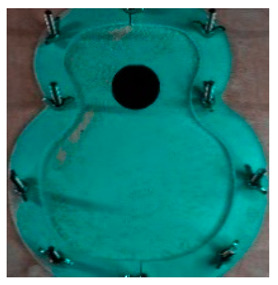
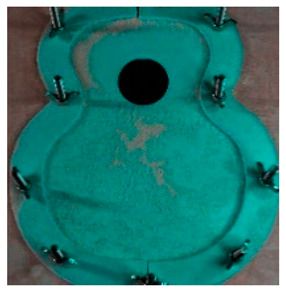
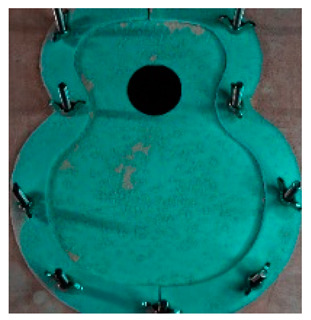
T02_81 printing angle 90°					
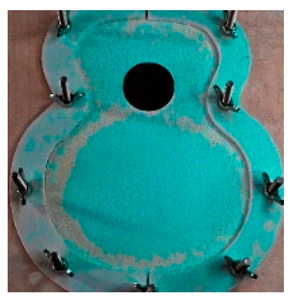
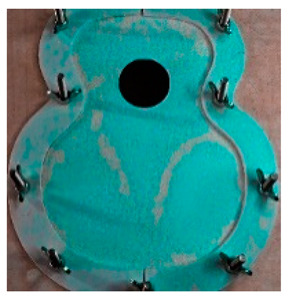
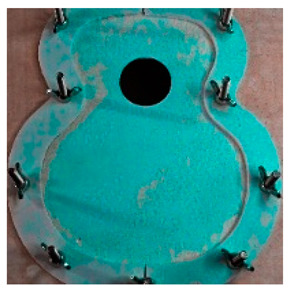
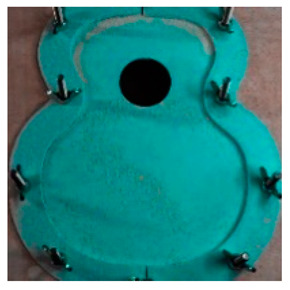
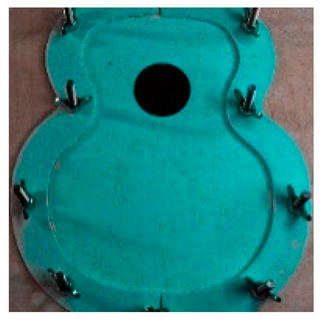
T02_81 printing angle +45°					
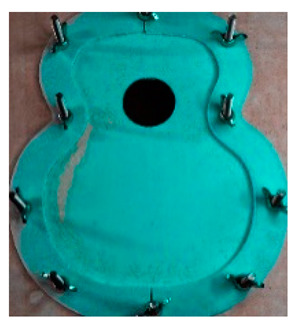
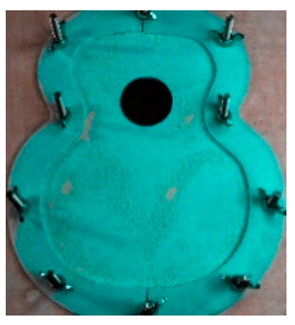
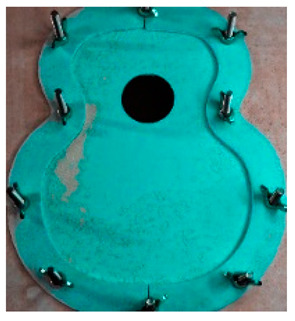
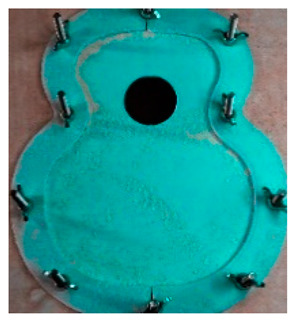
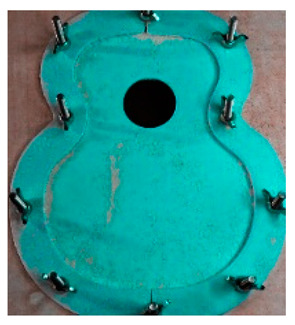
T02_81 printing angle +45°					
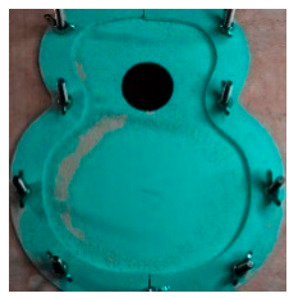
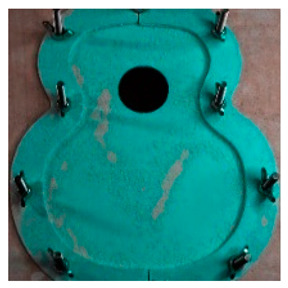
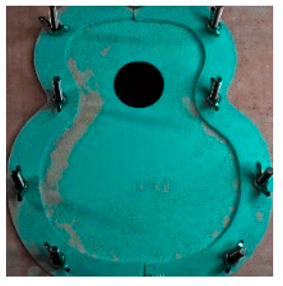
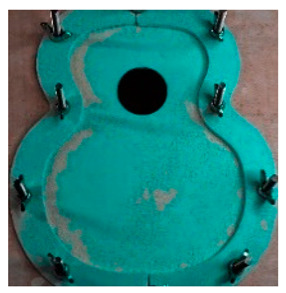
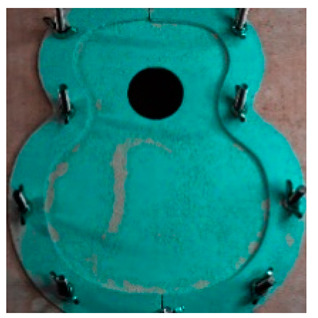
T02_81 printing angle −45°					
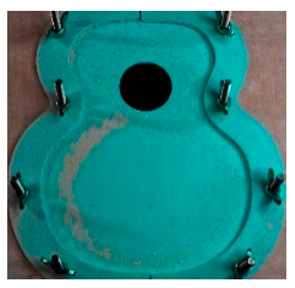
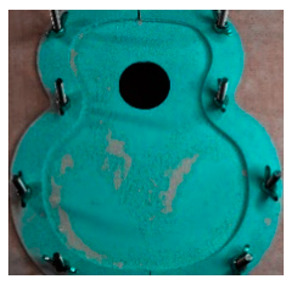
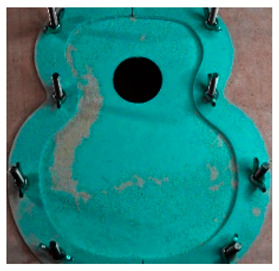
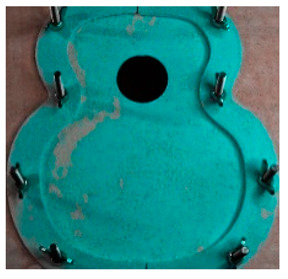
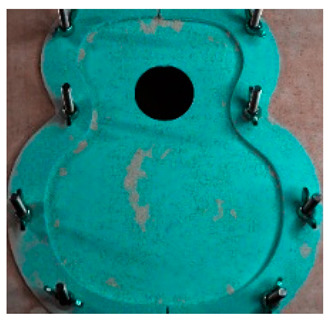
T02_81 printing angle −45°					
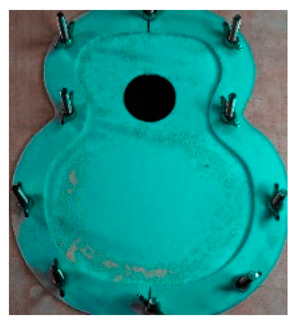
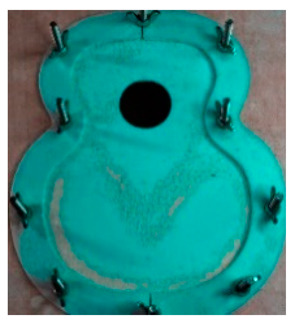
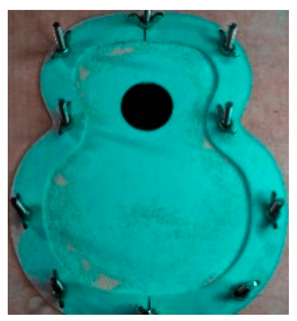
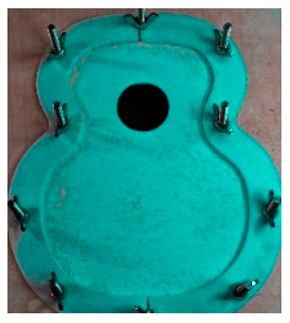
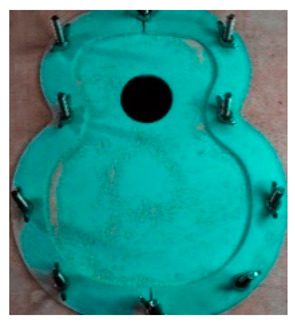
T02_81 printing angle ±45°					
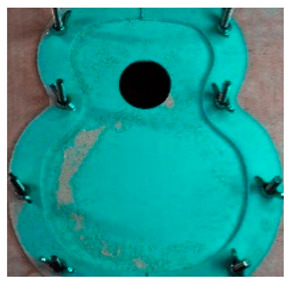
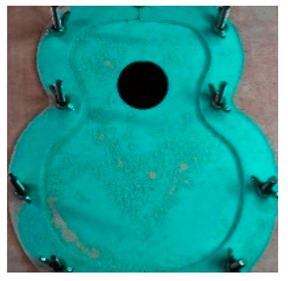
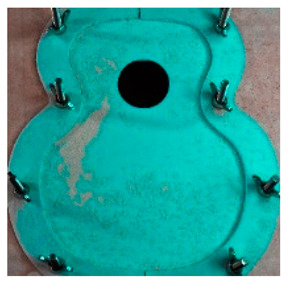
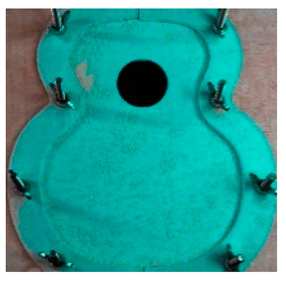
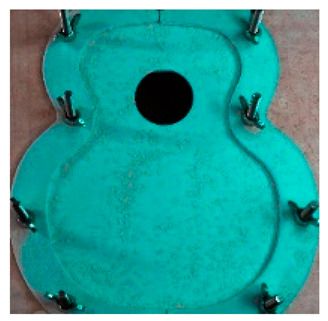
T02_81 printing angle ±45°					
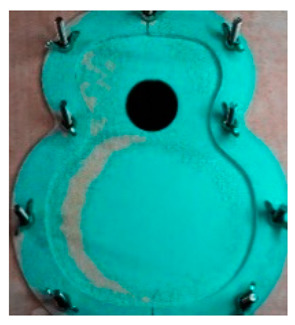
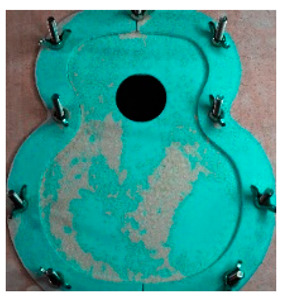
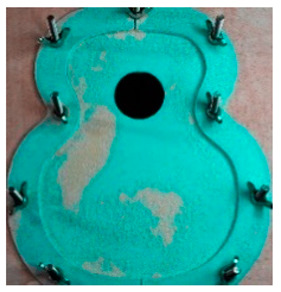
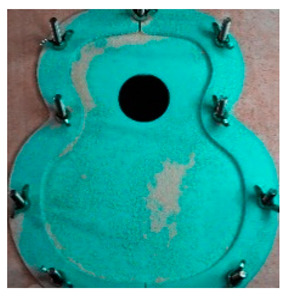
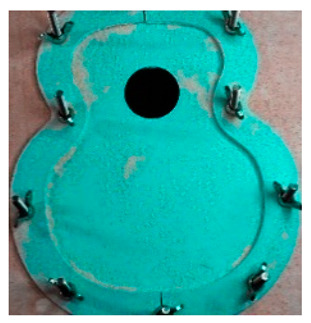

**Table A5:** Vibration patterns T01_99.

Modes	M1	M2	M3	M4	M5
FEM					
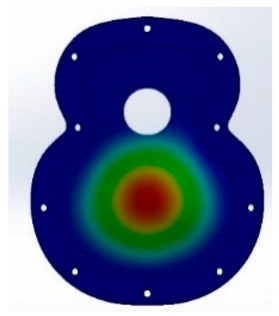
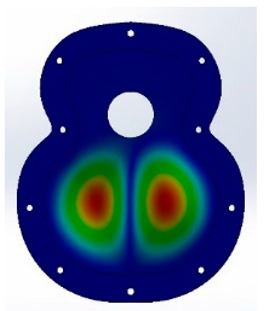
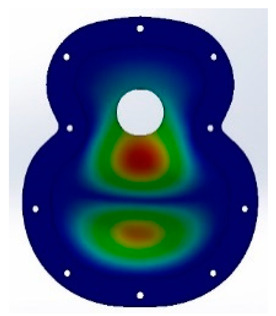
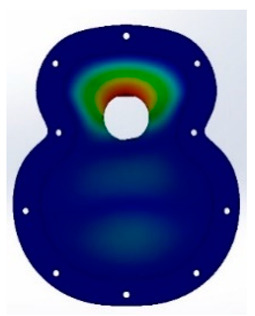
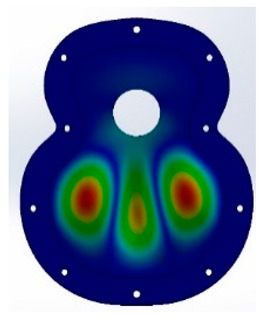
T01_99 printing angle 0°					
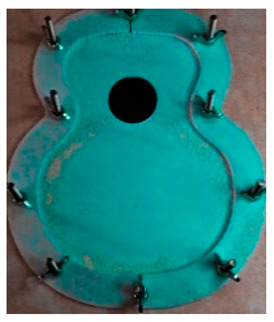
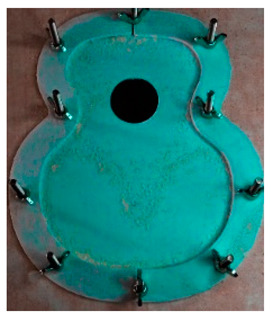
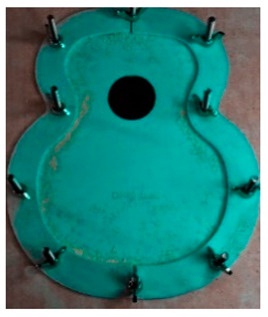
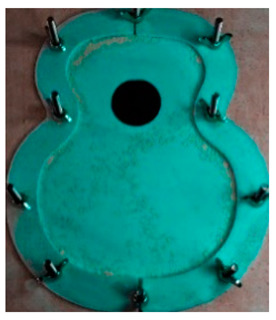
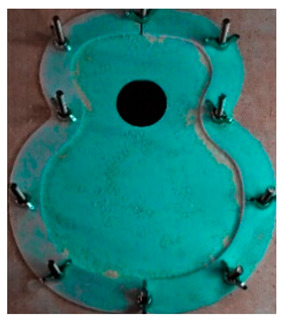
T01_99 printing angle 0°					
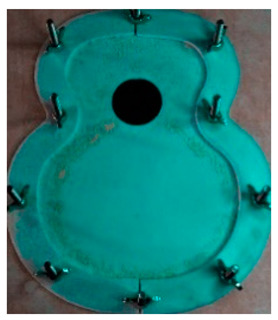
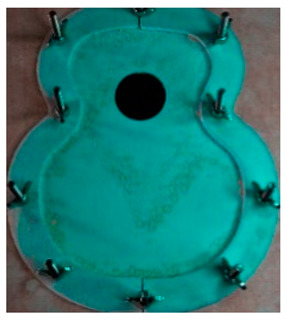
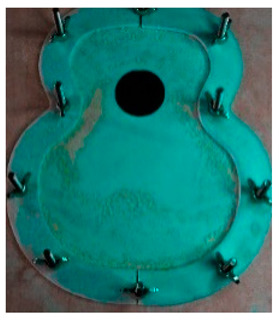
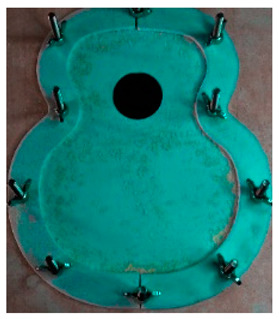
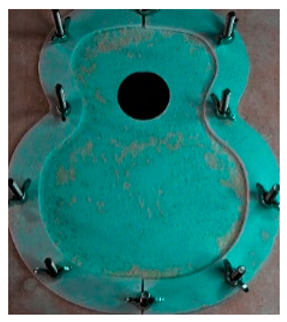
T01_99 printing angle 90°					
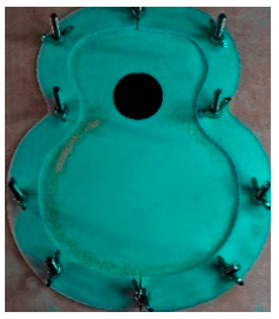
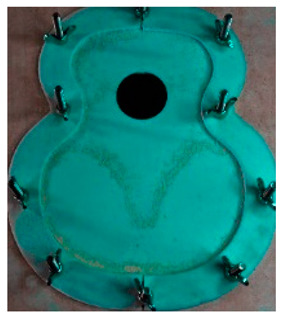
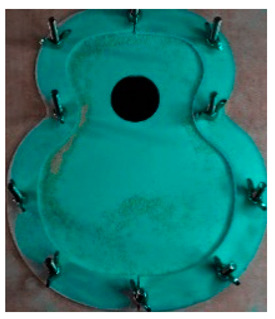
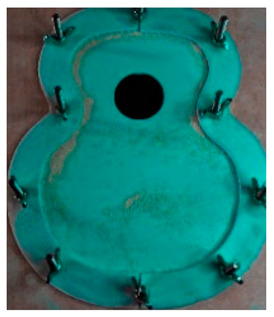
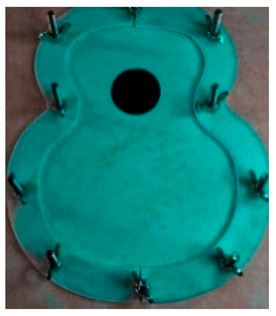
T01_99 printing angle 90°					
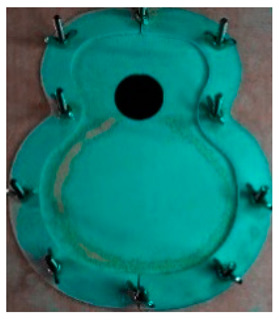
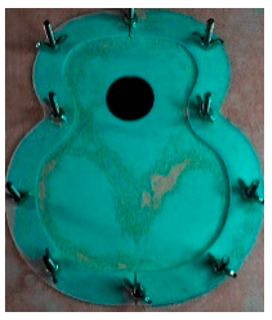
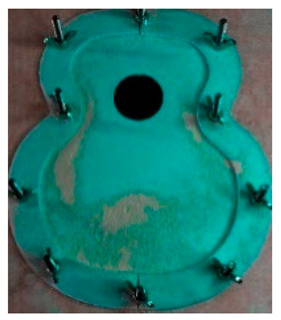
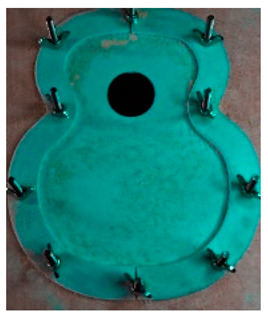
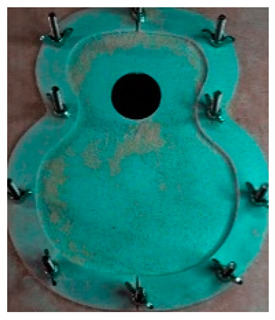
T01_99 printing angle +45°					
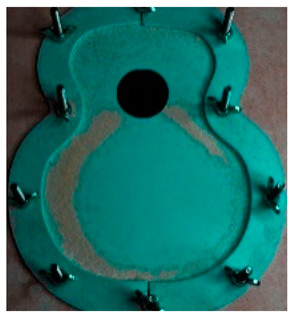
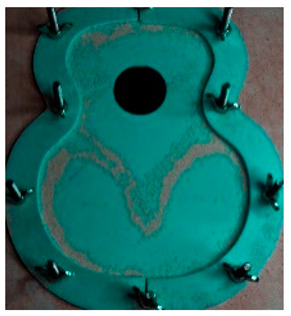
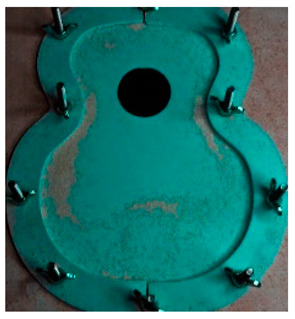
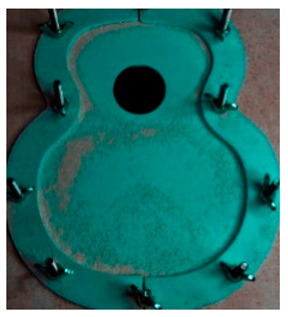
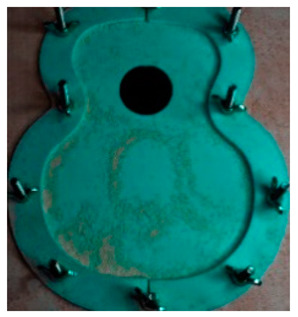
T01_99 printing angle +45°					
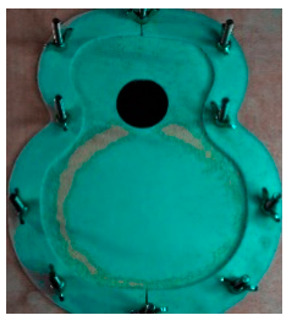
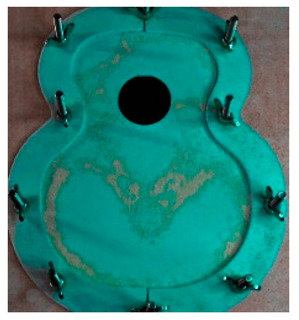
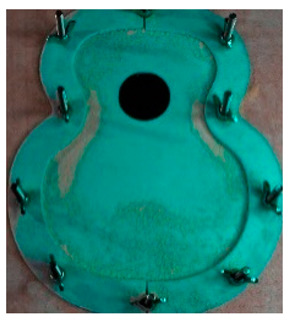
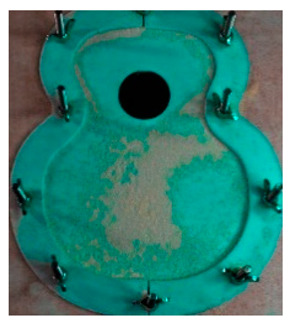
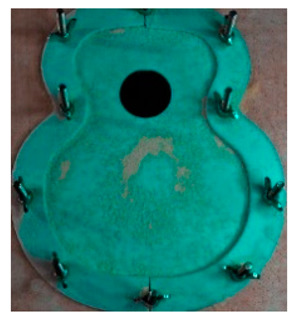
T01_99 printing angle −45°					
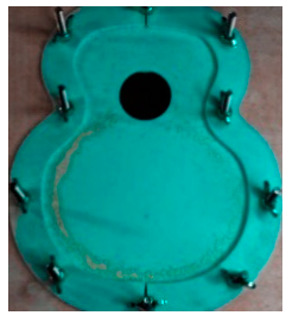
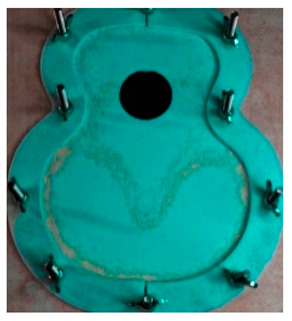
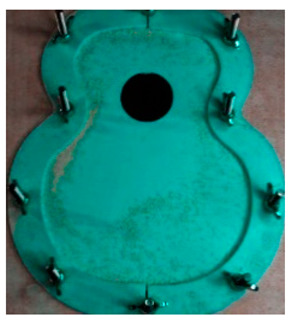
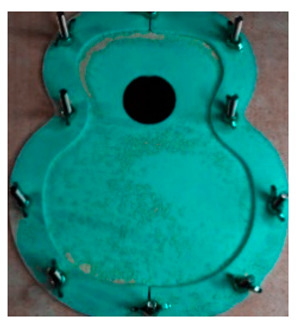
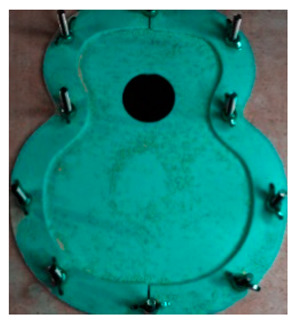
T01_99 printing angle −45°					
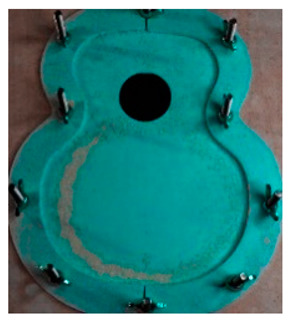
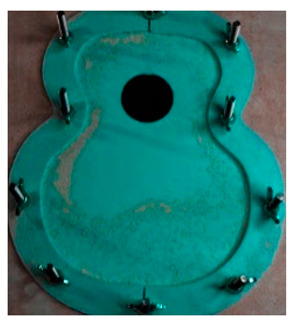
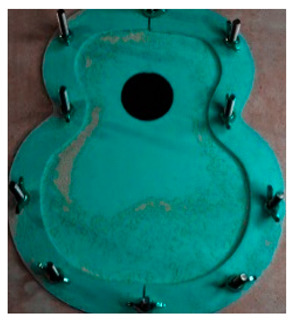
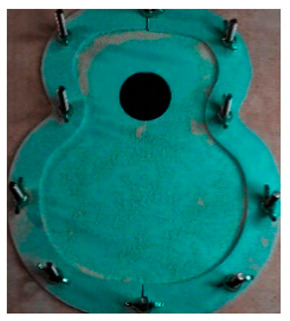
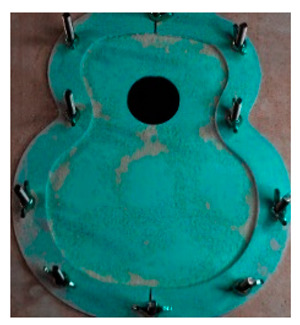
T01_99 printing angle ±45°					
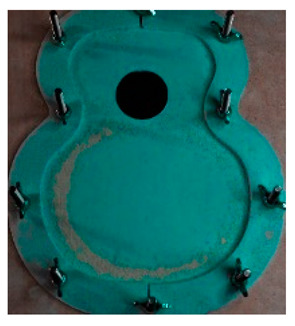
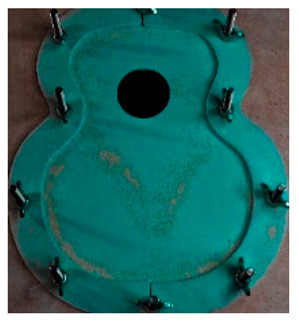
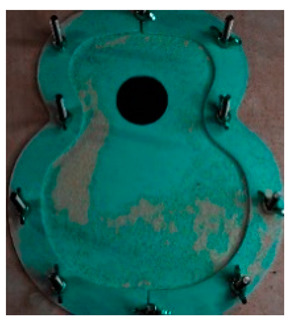
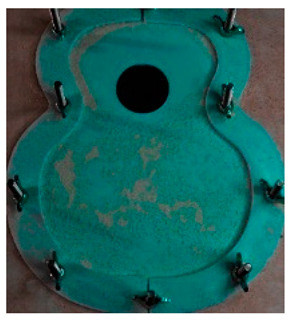
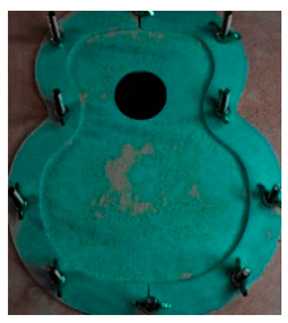
T01_99 printing angle ±45°					
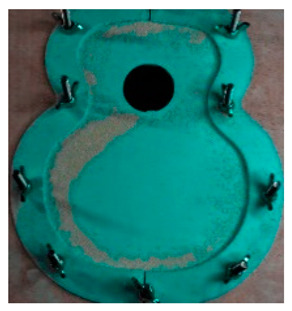
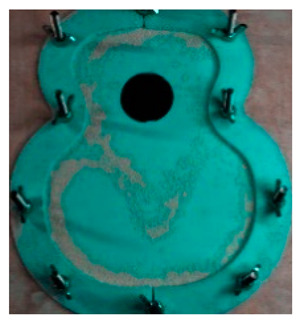
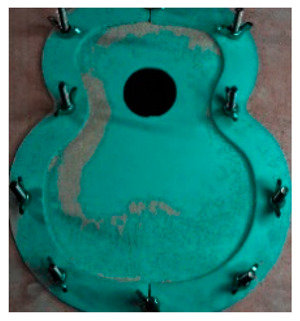
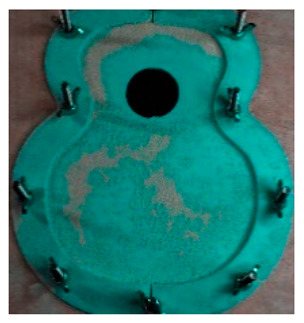
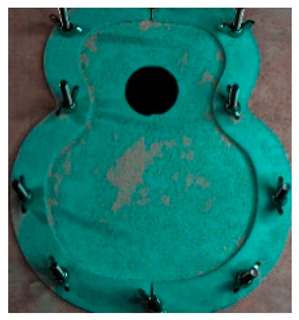

**Table A6:** Vibration patterns T02_99.

Modes	M1	M2	M3	M4	M5
FEM					
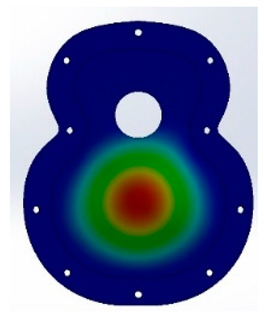
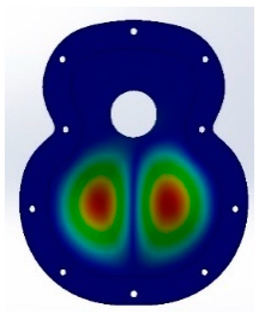
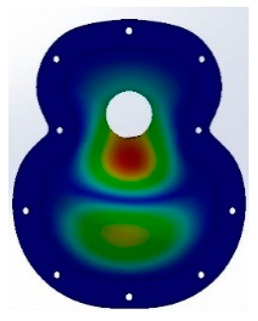
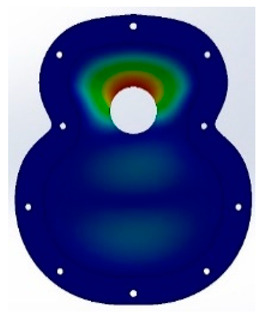
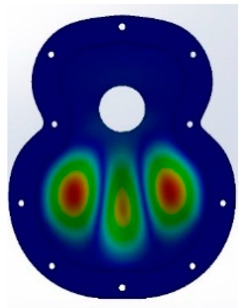
T02_99 printing angle 0°					
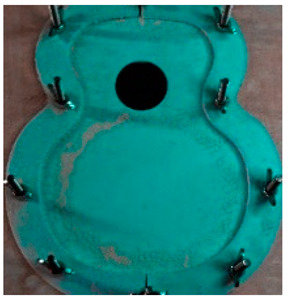
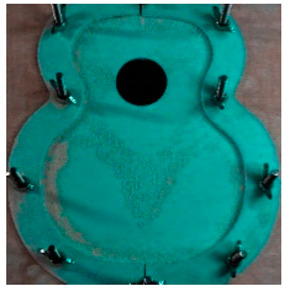
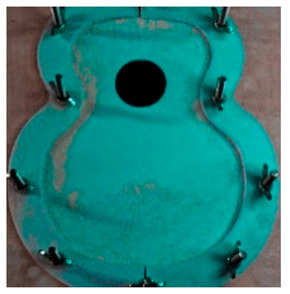
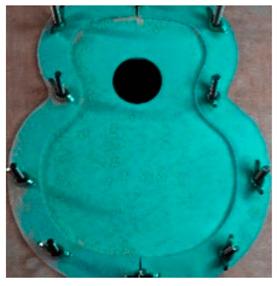
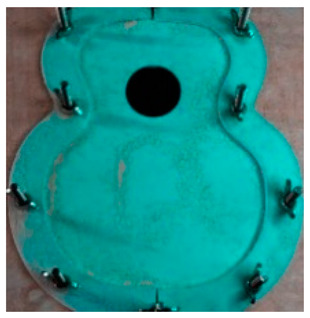
T02_99 printing angle 0°					
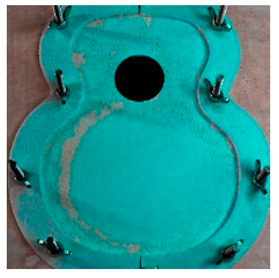
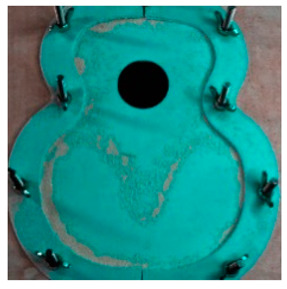
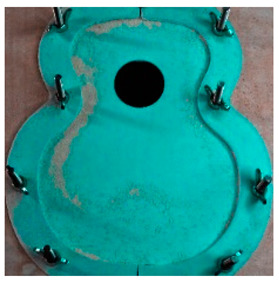
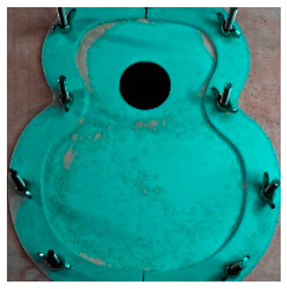
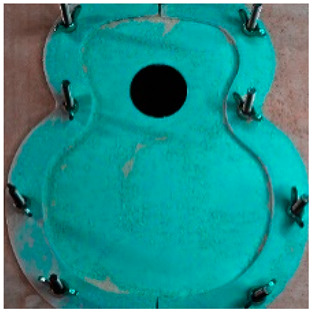
T02_99 printing angle 90°					
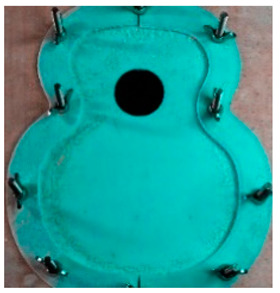
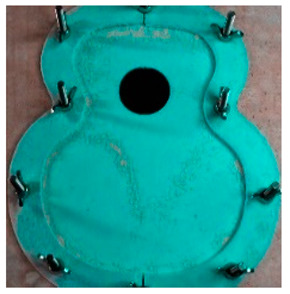
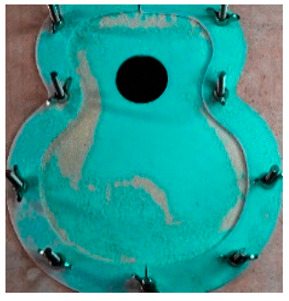
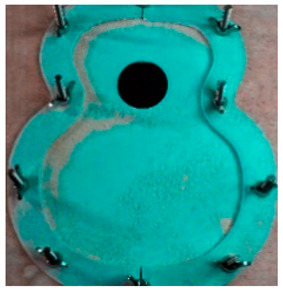
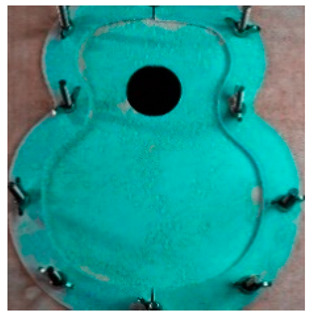
T02_99 printing angle 90°					
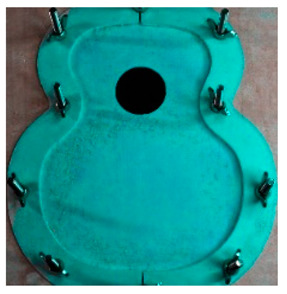
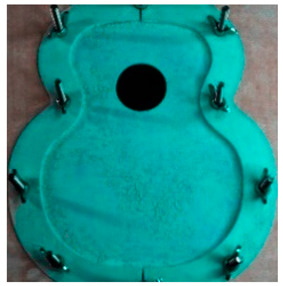
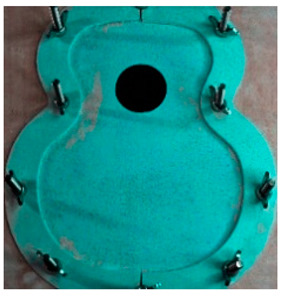
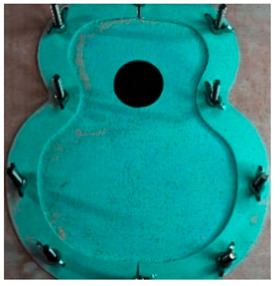
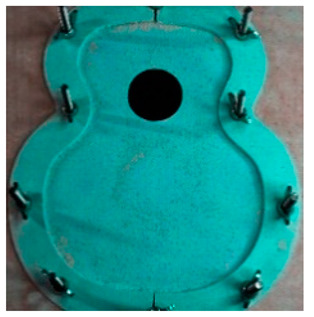
T02_99 printing angle +45°					
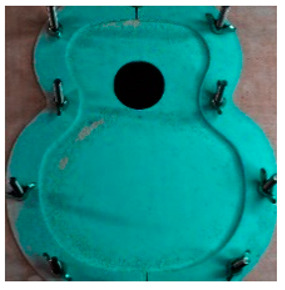
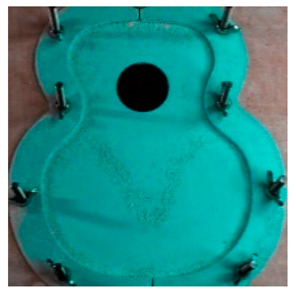
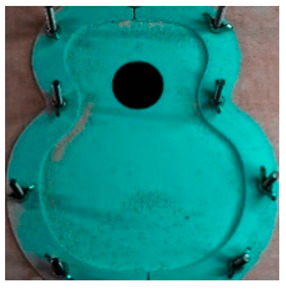
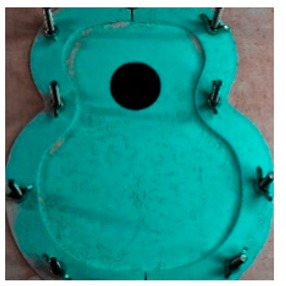
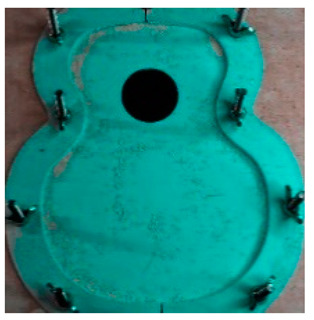
T02_99 printing angle +45°					
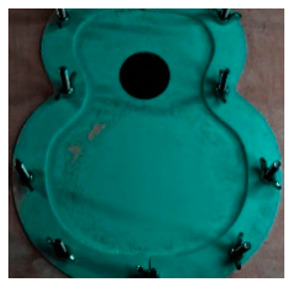
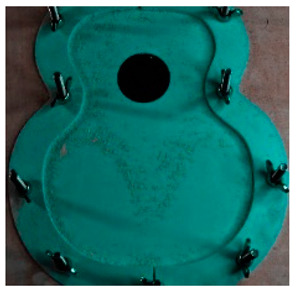
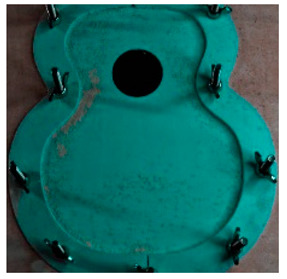
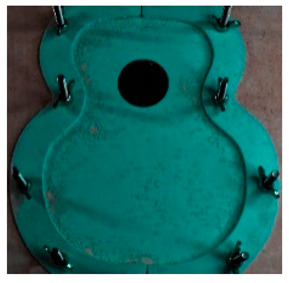
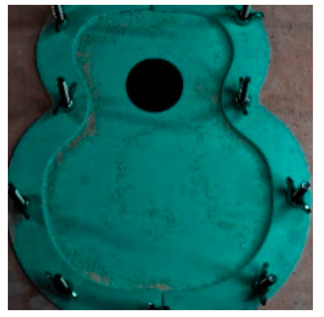
T02_99 printing angle −45°					
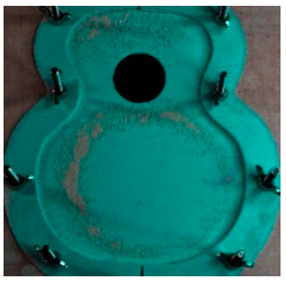
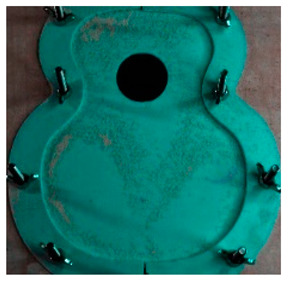
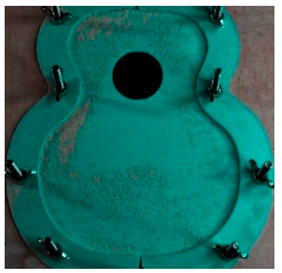
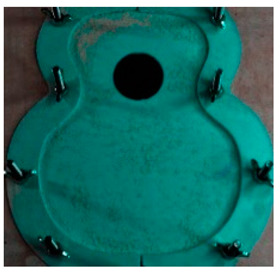
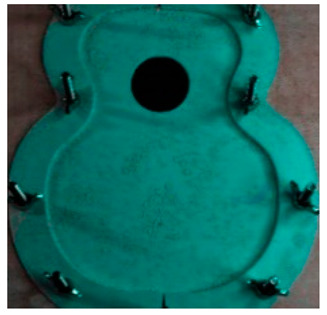
T02_99 printing angle −45°					
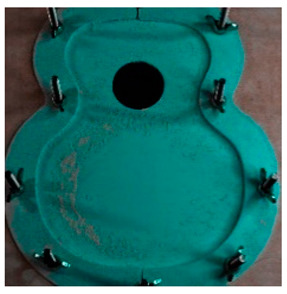
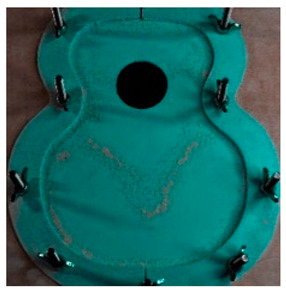
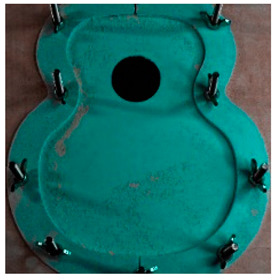
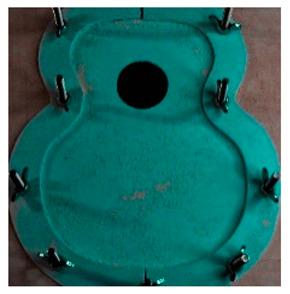
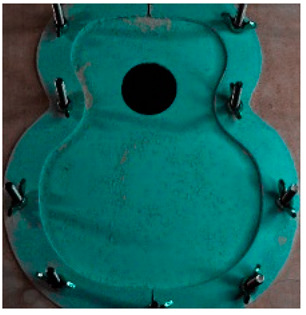
T02_99 printing angle ±45°					
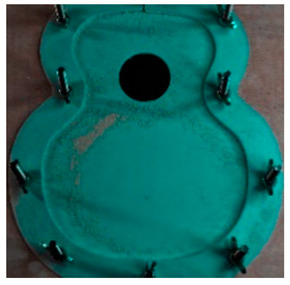
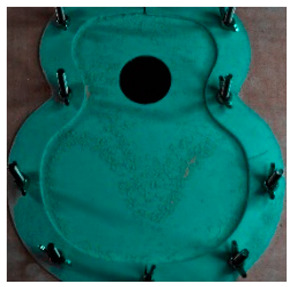
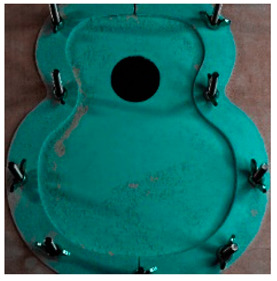
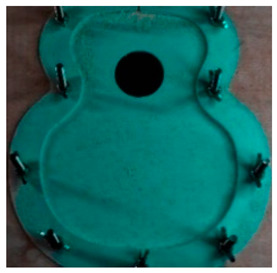
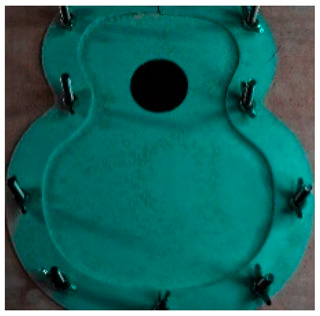
T02_99 printing angle ±45°					
